# Harnessing Human-Centered Design for Evidence-Based Psychosocial Interventions and Implementation Strategies in Community Settings: Protocol for Redesign to Improve Usability, Engagement, and Appropriateness

**DOI:** 10.2196/65446

**Published:** 2025-01-29

**Authors:** Aaron R Lyon, Sean A Munson, Michael D Pullmann, Brittany Mosser, Tricia Aung, John Fortney, Alex Dopp, Katie P Osterhage, Helen G Haile, Kathryn E Bruzios, Brittany E Blanchard, Ryan Allred, Macey R Fuller, Patrick J Raue, Ian Bennett, Jill Locke, Karen Bearss, Denise Walker, Elizabeth Connors, Eric Bruns, Jenna Van Draanen, Doyanne Darnell, Patricia A Areán

**Affiliations:** 1 Department of Psychiatry and Behavioral Sciences University of Washington Seattle, WA United States; 2 Department of Human Centered Design & Engineering University of Washington Seattle, WA United States; 3 Department of International Health Johns Hopkins Bloomberg School of Public Health Baltimore, MD United States; 4 Center of Innovation for Veteran-Centered and Value-Driven Care Health Services Research and Development Department of Veterans Affairs Seattle, WA United States; 5 RAND Santa Monica, CA United States; 6 Department of Family Medicine University of Washington, WA United States; 7 Department of Medicine UMass Chan Medical School Worcester, MA United States; 8 Veterans Affairs Bedford Healthcare System Bedford, MA United States; 9 Departments of Family Medicine University of Washington Seattle, WA United States; 10 Department of Global Health University of Washington Seattle, WA United States; 11 School of Social Work University of Washington Seattle, WA United States; 12 Department of Psychiatry and The Child Study Center Yale School of Medicine New Haven, CT United States; 13 Department of Child, Family, and Population Health Nursing University of Washington Seattle, WA United States; 14 Department of Health Systems and Population Health University of Washington Seattle, WA United States; 15 Division of Services and Interventions Research National Institute of Mental Health Bethesda, MD United States

**Keywords:** implementation science, human-centered design, evidence-based psychosocial interventions, mental health

## Abstract

**Background:**

Although substantial progress has been made in establishing evidence-based psychosocial clinical interventions and implementation strategies for mental health, translating research into practice—particularly in more accessible, community settings—has been slow.

**Objective:**

This protocol outlines the renewal of the National Institute of Mental Health–funded University of Washington Advanced Laboratories for Accelerating the Reach and Impact of Treatments for Youth and Adults with Mental Illness Center, which draws from human-centered design (HCD) and implementation science to improve clinical interventions and implementation strategies. The Center’s second round of funding (2023-2028) focuses on using the Discover, Design and Build, and Test (DDBT) framework to address 3 priority clinical intervention and implementation strategy mechanisms (ie, usability, engagement, and appropriateness), which we identified as challenges to implementation and scalability during the first iteration of the center. Local redesign teams work collaboratively and share decision-making to carry out DDBT.

**Methods:**

All 4 core studies received institutional review board approval by June 2024, and each pilot project will pursue institutional review board approval when awarded. We will provide research infrastructure to 1 large effectiveness study and 3 exploratory pilot studies as part of the center grant. At least 4 additional small pilot studies will be solicited and funded by the center. All studies will explore the use of DDBT for clinical interventions and implementation strategies to identify modification targets to improve usability, engagement, and appropriateness in accessible nonspecialty settings (Discover phase); develop redesign solutions with local teams to address modification targets (Design and Build phase); and determine if redesign improves usability, engagement, and appropriateness (Test phase), as well as implementation outcomes. Center staff will collaborate with local redesign teams to develop and test clinical interventions and implementation strategies for community settings. We will collaborate with teams to use methods and centerwide measures that facilitate cross-project analysis of the effects of DDBT-driven redesign on outcomes of interest.

**Results:**

As of January 2025, three of the 4 core studies are underway. We will generate additional evidence on the robustness of DDBT and whether combining HCD and implementation science is an asset for improving clinical interventions and implementation strategies.

**Conclusions:**

During the first round of the center, we established that DDBT is a useful approach to systematically identify and address chronic challenges of implementing clinical interventions and implementation strategies. In this subsequent grant, we expect to increase evidence of DDBT’s impact on clinical interventions and implementation strategies by expanding a list of common challenges that could benefit from modification, a list of exemplary solutions to address these challenges, and guidance on using the DDBT framework. These resources will contribute to broader discourse on how to enhance implementation of clinical interventions and implementation strategies that integrate HCD and implementation science.

**International Registered Report Identifier (IRRID):**

PRR1-10.2196/65446

## Introduction

### Background

Psychosocial clinical interventions such as psychotherapy, counseling, and case management are a preferred mode of treatment by most people seeking care for mental health problems [1-5]. Access to evidence-based clinical interventions remains variable among diverse groups, leading to mental health disparities across racial and ethnic, geographic, and socioeconomic status [6-16]. Furthermore, implementing clinical interventions in nontraditional and integrated settings (eg, primary care, telehealth platforms, and schools) has shown mixed success. These settings can serve as a *safety net* for accessing mental health treatment when traditional options are inaccessible [17-20]. Addressing barriers to implementing clinical interventions in these settings is vital to promote more equitable access to mental health services for all.

Poor availability of clinical interventions is often due to intervention complexity and suboptimal fit with many settings where clinical interventions are deployed [21]. Implementation strategies—“systematic intervention process(es) to promote the uptake of evidence-based health innovations into usual care”—have often taken the form of complex tools and processes (eg, train the trainer, booster training, incentive models, and decision supports) [22,23]. However, these often fall short because they can be excessively costly and cumbersome [22,24]. Different needs of recipients and settings can lead to high rates of reactive adaptations of clinical interventions and implementation strategies by their intended users in many settings where they are deployed. Reactive adaptations are unplanned or improvised changes during an implementation process in response to unanticipated challenges [25]. While reactive adaptations can compromise clinical potency, those that are proactively tailored to different care settings can improve sustainability and impact [26-29]. A systematic review of cultural adaptations to health and mental health services highlighted how adaptations motivated by cultural sensitivity are not guaranteed to demonstrate increased efficacy [30]. Instead, patient-centered approaches that account for individualized needs and barriers to service are recommended to guide adaptations [30,31].

### Human-Centered Design and Implementation Science

Human-centered design (HCD) and the closely related discipline user-centered design offer a suite of methods to develop useful, compelling, intuitive, and enjoyable products, services, and tools based on people’s needs [32,33]. HCD relates to the evolution of human-computer interaction (HCI), a multidisciplinary field that incorporates computer science, cognitive science, and human factors engineering as a response to personal computing, collaborative work, and interconnected technologies in everyday life [34]. While HCD’s origins are rooted in technology, it has been used beyond the context of digital technologies to address therapeutic elements and implementation supports [35]. HCD has been applied to improve usability, reduce burden, and increase the contextual appropriateness of clinical interventions and implementation strategies [36-40].

The fields of HCD and implementation science share common objectives and offer complementary methods that can support clinical interventions and implementation strategies innovation and redesign [41-44]. HCD techniques are particularly well positioned to help with redesign, which we define as adaptations to clinical interventions and implementation strategies while preserving effective components (ie, fidelity-consistent adaptation) [45]. HCD’s traditions of situating problem discovery and solutions in user needs, usability, engagement, innovation, and rapid exploration are core strengths that align with implementation science’s goal of improved adoption, fidelity, reach, and adaptation of clinical interventions and implementation strategies [41]. Combining HCD and implementation science traditions for clinical intervention and implementation strategy redesign grounds novelty in empirical evidence.

### Integrating HCD and Implementation Science Through the Discover, Design and Build, and Test Framework

**Figure 1:**
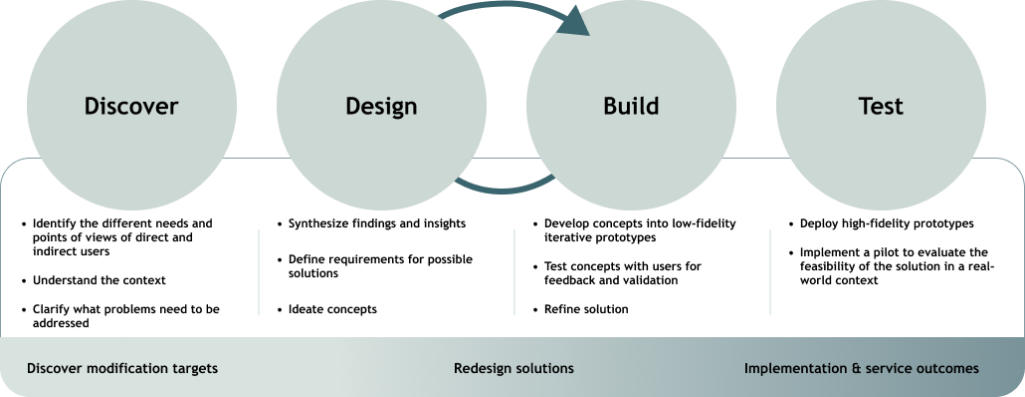
Discover, Design and Build, and Test (DDBT) redesign framework.

The University of Washington Advanced Laboratories for Accelerating the Reach and Impact of Treatments for Youth and Adults with Mental Illness Center (UWAC), which is funded by a grant from the National Institute of Mental Health (NIMH), is a multidisciplinary team of experts from the fields of mental health, implementation science, and HCD focused on improving usability, engagement, and appropriateness of clinical interventions and implementation strategies in diverse and nonspecialty settings (eg, rural, urban, low-income, primary care, and schools). Drawing on strengths from different disciplines, UWAC developed the Discover, Design and Build, and Test (DDBT) framework (Figure 1) at the start of the center (ie, “UWAC 1.0”). The current DDBT framework guides teams in redesigning clinical interventions and implementation strategies to improve usability, engagement, appropriateness, and implementation outcomes while preserving clinical interventions’ core components [46]. Key principles underlying this model include the following: (1) not all clinical interventions and implementation strategies are designed for all settings; (2) “there is no implementation without adaptation” [47]; (3) unchecked, reactive adaptations have the potential to exclude essential active ingredients [48,49]; and (4) reactively adapted clinical interventions and implementation strategies can negatively impact implementation and clinical outcomes.

**Figure 2:**
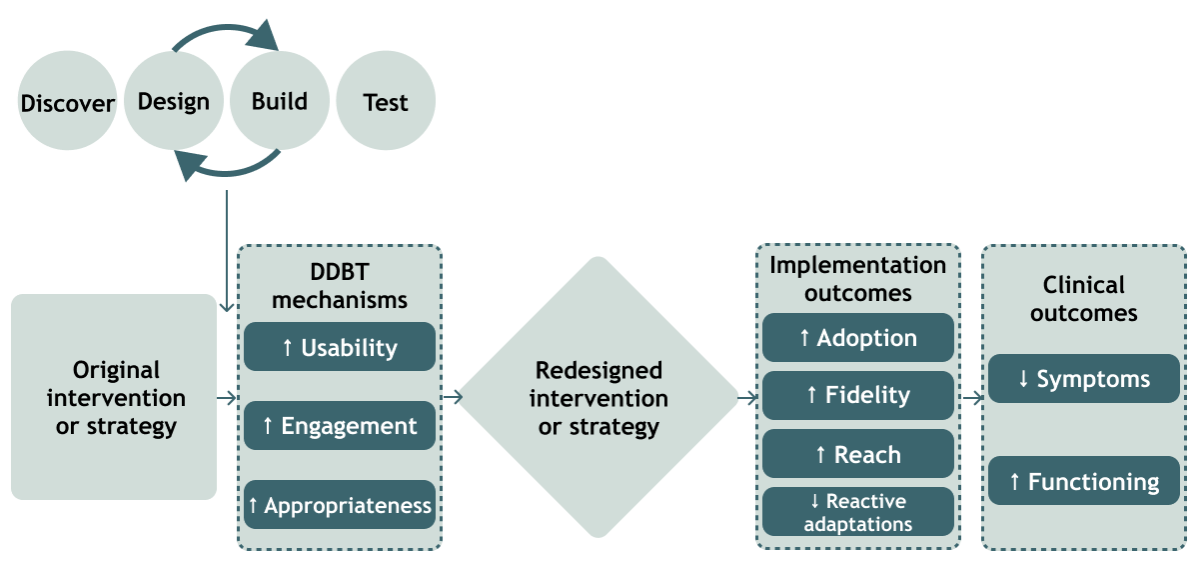
University of Washington Advanced Laboratories for Accelerating the Reach and Impact of Treatments for Youth and Adults with Mental Illness Center (UWAC) theory of change for clinical intervention and implementation strategy redesign. DDBT: Discover, Design and Build, and Test.

The DDBT framework is modeled after existing HCD frameworks [50] and is an iterative stepped approach to systematically (1) understand usability constraints of existing clinical interventions and implementation strategies, (2) iteratively design solutions for usability challenges with redesign teams of direct and indirect users, and (3) test and refine prototypes. We define direct users (also known as “primary users”) as people who directly interact with the clinical intervention and implementation strategy and indirect users (also known as “secondary users”) as people affected by the clinical intervention and implementation strategy. HCD places a strong emphasis on explicitly identifying relevant community collaborators and users to ensure that new products effectively meet their needs [51,52]. DDBT starts by identifying multilevel factors that drive clinical intervention and implementation strategy usability problems, engagement challenges, and problems with contextual appropriateness (Discover phase). Once problems and challenges are identified, modifications are iteratively created between the design team and practitioners and clients, until a new version of the clinical interventions and implementation strategies is developed to address crucial issues and enhance usability, engagement, and appropriateness (Figure 2; Design and Build phase). Early prototypes of clinical interventions and implementation strategies are assessed with small samples (eg, 5-25 participants) to answer design questions using paper or other “low-fidelity” (ie, sufficient to communicate a concept but potentially lacking functionality, some content, and look and feel of final materials) versions of modifications, which reduces waste of unnecessary investment in programming and development until as late in the process as possible. Findings from the Design and Build phase are incorporated to develop high-fidelity prototypes, which are tested against the unadapted version to ascertain if the modified clinical interventions and implementation strategies result in improved implementation (eg, increased adoption, fidelity, reach, and reduced reactive adaptations) and equivalent or better mental and behavioral health outcomes because of the changes to usability, engagement, and appropriateness (Test phase). Additional details on the DDBT framework are outlined in our UWAC 1.0 protocol paper [46].

All UWAC research uses the DDBT framework, which is applied flexibly based on project needs and allows us to evaluate the extent to which incorporating HCD and implementation science methods impacts clinical interventions and implementation strategies. Since 2018, DDBT has been used in >18 UWAC studies and 16 National Institutes of Health (NIH)–funded awards external to UWAC. During UWAC 1.0, we originally assessed impact of DDBT on 3 mechanisms: learnability (ie, extent to which users can understand or facilitate use) [46], usability (ie, extent to which users can achieve specified goals of effectiveness, efficiency, and satisfaction) [50], and sustained quality of care (ie, extent of treatment fidelity and impact on target outcomes) [46]. Analysis of UWAC 1.0 projects resulted in (1) identification of common usability issues in clinical interventions and implementation strategies that could benefit from modification (ie, “typology of modification targets”) and corresponding heuristics to guide their design [53], (2) reflections on potential exemplary solutions to these challenges (ie, library of clinical interventions and implementation strategies redesign solutions), and (3) guidelines for using the DDBT framework [46].

### UWAC Theory of Change for Clinical Intervention and Implementation Strategy Redesign

Our UWAC 1.0 findings and the implementation literature [21] outline how limited availability and the use of clinical interventions and implementation strategies are persistently attributable to organizational and system characteristics (eg, readiness to adopt, resources and culture, and leadership), clinician and adopter characteristics (eg, appropriateness and perceived efficacy of clinical interventions and implementation strategies for patients) [28,54,55], and incentives to engage in clinical interventions and implementation strategies [29,56].

As a result, we updated DDBT’s underlying theory of change to highlight how adoption of clinical interventions and implementation strategies are largely due to usability (eg, extent to which clinical interventions and implementation strategies can be used to achieve specified goals with effectiveness, efficiency, and satisfaction) [53]; engagement (eg, degree of user participation and enthusiasm for the aspects of clinical interventions and implementation strategies that require user involvement) [57]; and appropriateness (eg, perceived fit, relevance, or compatibility of clinical interventions and implementation strategies for a given practice setting, practitioner, or consumer) [58] (Figure 2). These mechanisms are direct targets of DDBT-driven redesign and the focus of the new iteration of funding between 2023 and 2028 (ie, “UWAC 2.0”). We ultimately expect DDBT to result in changes to proximal implementation outcomes (adoption, fidelity, reach, and reactive adaptation) and clinical outcomes.

### DDBT Mechanisms

#### Usability

Usability is an underlying outcome at all stages of the HCD process. Understanding the extent to which designs are unusable and opportunities to increase an existing solution’s usability can inspire innovation and adoption [53]. Deployment of clinical interventions and implementation strategies will continue to be subpar unless usability can be addressed, the historically unidirectional relationship between developers and users can be overcome, and insufficient incorporation of user perspectives can be remedied [59]. Usability is assessed through usability evaluations and usability testing, where prototypes are evaluated using established heuristics [60] and observing users complete critical tasks [61]. Usability assessment methods stem from evaluating technologies; however, these techniques have been used to improve usability, decrease burden, and increase contextual appropriateness of nontechnological mental health clinical interventions and implementation strategies [5,36,39,53]. Approaches to assessing usability frequently couple interview-type questions with surveys and observation [62]. As a form of usability evaluation, metrics such as the System Usability Scale (SUS) [63] are questionnaires that assess perceived usability. The SUS is a widely used instrument to measure usability of technologies by industry. Task-based usability testing involves asking participants to complete tasks while using a prototype or product. This method can be used to gather baseline usability data for an existing clinical intervention and implementation strategy and assess usability of clinical intervention and implementation strategy prototypes [53]. The think-aloud protocol (TAP) involves participants verbalizing thought processes as they use clinical interventions and implementation strategies [39] to complete assigned tasks, including actions they consider taking and reactions to materials as they encounter them. Similarly, the Cognitive Walkthrough for Implementation Strategies (CWIS) is a 6-step method for evaluating clinical interventions and implementation strategies usability, which can include interviews as part of task-based usability testing [39].

UWAC 1.0 projects assessed usability through usability questionnaires, CWIS [39], and TAP [64,65]. We developed the Intervention Usability Scale (IUS) [37] and Implementation Strategy Usability Scale (ISUS) [39] to better measure clinical intervention and implementation strategy usability; these scales are closely aligned with SUS. SUS scores of ≥70 out of 100 are considered adequate usability, and we anticipate a similar threshold for IUS and ISUS. Cross-project usability data informed the typology of modification targets: 12 unique categories of clinical intervention and implementation strategy usability issues of varying severity. These categories help researchers understand common barriers to clinical intervention and implementation strategy use that can be prevented and addressed during clinical intervention and implementation strategy redesign [53].

#### Engagement

Engagement, and adaptations to improve engagement, relates to clinical interventions fidelity and clinical outcomes [66-69] and is a defining feature of quality of care [70-72]. Engagement describes user connection to clinical interventions and implementation strategies and their capacity to sustain a connection [57]. Engagement is distinct from common health concepts of compliance, adherence, and coverage because it incorporates a dimension of quality and welcomes the possibility that different people may engage differently with different parts of clinical interventions and implementation strategies based on varying needs. Typical users include the practitioners who deliver them (eg, clinicians, implementation practitioners, and intermediaries) and the individuals who receive them (eg, clients, practitioners, and service system administrators). Our work has found that engagement can be negatively impacted by insufficient buy-in, components that are inaccessible to different users, little support for communication or rapport building, and requirements or constraints that inadvertently shift one user’s responsibilities to another (eg, practitioners completing client tasks) [53].

Engagement is a common focus in HCI literature [57,73-75], where there is an active conversation around how to best assess the quality of interactions rather than quantity of interactions. Multidisciplinary UWAC project teams present an opportunity to incorporate different approaches to improving engagement in translational research [76-78]. We conceptualize engagement as a multifaceted construct focused on interaction quality (ie, participation and enthusiasm) that is enhanced by clinical interventions and implementation strategies that are well designed and result in improved adoption, fidelity, reach, and adaptations. There are subjectivity-oriented and objectivity-oriented approaches to measuring engagement [57]. Subjectivity-oriented measures are self-reported and include questionnaires, observation, perceived task outcomes, and interviews. Objectivity-oriented measures minimize researcher involvement and can include behavior logging, psychophysiological measurements, or telemetry. Within HCI, objectivity-oriented measures such as user data—logs, time, number of interactions, and frequency of log-ins—are commonly used.

#### Appropriateness

Appropriateness captures perceived fit between clinical interventions and implementation strategies and the settings to which they are deployed [58]. Client needs and therapeutic style drive how mental health practitioners modify clinical interventions and implementation strategies in practice [26]. During UWAC 1.0, we observed how practitioners and recipients felt that exciting innovations can be inappropriate for specific contexts (eg, schools) or for users facing challenges with identifying and selecting goals or implementation plans. Challenges included clinical interventions and implementation strategies’ excessive time demands in their delivery, incompatibility with existing workflows or roles, unavailable system infrastructure requirements, overreliance on digital technology, and, importantly, practitioner perceptions of the fit of the clinical interventions and implementation strategies to specific client problem types. These issues are well documented as multifaceted factors that influence clinical intervention and implementation strategy adaptations [29,45]. During UWAC 1.0, we used the Intervention Appropriateness Measure (IAM) [79] across projects to assess appropriateness.

### Study Purpose

#### Center Aims and Structure

Our goal is to overcome obstacles that prevent quality mental health interventions from reaching historically marginalized groups through addressing critical clinical intervention and implementation strategy problems with the DDBT framework. Building on learnings during the first iteration of the center, UWAC 2.0 focuses on addressing longstanding problems with *usability*, *engagement*, and *appropriateness* of clinical interventions and implementation strategies that result in high rates of reactive adaptations in settings where they are deployed. Within UWAC, the Methods Core team provides methodological and technical support to all projects and maintains cross-cutting project data on UWAC outcomes to determine the impact of DDBT on clinical interventions and implementation strategies. These data will be used to refine and expand UWAC’s typology of modification targets and library of redesign solutions. This iteration of the center emphasizes increased leadership and application of DDBT methods by local project redesign teams (eg, administrators or champions) that receive methodological training and supports from the Methods Core team to work alongside investigators, increasing their decision-making at all stages of the design process. This is a shift from the previous centralized model, where projects engaged users, but project principal investigators (PIs) and UWAC Methods Core team members often led the design process. The Methods Core team will address the subsequent aims during UWAC 2.0.

#### Aim 1: Identify Clinical Intervention and Implementation Strategy Modification Targets to Improve Usability, Engagement, and Appropriateness in Accessible Nonspecialty Settings (Discover Phase)

Known determinants of successful clinical intervention and implementation strategy use exist at several levels, including clinical interventions (eg, complexity); practitioner and client (eg, training, attitudes, and intention to use); and organizational (eg, climate, leadership, resources, and supervision). Historically, adaptations of clinical interventions and implementation strategies have been driven by academics rather than the lived experiences of recipients. Using the Consolidated Framework for Implementation Research (CFIR) [80] as our guide in the Discover phase of each UWAC project, we support project redesign teams to use HCD methods to identify targets within our typology of modification targets [53]. Qualitative analyses will allow us to compare targets identified via local DDBT, characterized by user involvement in redesign teams and shared decision-making about target prioritization and solutions, with those derived from our original, centralized application of DDBT [46]. Aim 1 outcomes will inform typology revisions and allow for comparisons between the original, centralized DDBT and the local DDBT. An updated typology will be broadly disseminated to inform future research.

#### Aim 2: Develop Redesign Solutions With Local Teams to Address Clinical Intervention and Implementation Strategy Modification Targets (Design and Build Phase)

Using rapid, iterative design principles, we are supporting research project teams in redesigning clinical interventions and implementation strategies to enhance usability, engagement, and appropriateness. We will systematically catalog these design solutions using the Framework for Modifications and Adaptations of Evidence-Based Interventions (FRAME) or FRAME for Implementation Strategies (FRAME-IS) [26,29,45]. We will examine solutions and the populations, organization types or structures, practitioner types, and clinical interventions and implementation strategies in which they work, identifying solutions that transfer across different contexts or are uniquely suited to specific contexts. Aim 2 outcomes will be compared with centralized DDBT outputs, resulting in an updated library of redesign solutions organized by target and redesign method (ie, localized vs centralized), and shared with the interested community.

#### Aim 3: Determine If Redesign Affects Changes in Usability, Engagement, and Appropriateness (Test Phase)

Each project conducting a Test phase will include a hybrid effectiveness-implementation trial [81,82] with a primary comparison between the original clinical intervention and implementation strategy and the DDBT-adapted version on theorized mechanisms (usability, engagement, and appropriateness), implementation outcomes (adoption, fidelity, reach, and adaptations), and client outcomes. Projects will apply FRAME or FRAME-IS [29,45] to examine the extent to which DDBT decreases reactive adaptations to the clinical intervention and implementation strategy (ie, unplanned or due to unanticipated obstacles) during implementation. We hypothesize that DDBT-informed, prospective, and planned adaptations will reduce the number and extent of reactive adaptations. The Methods Core team will systematically integrate new data from the UWAC projects with existing data in an integrated cross-project analysis of how redesign affects theorized mechanisms, implementation outcomes, and patient outcomes. Aim 3 outcomes will be disseminated to the field and inform new projects designed to test which redesign strategies best improve DDBT mechanisms and outcomes.

## Methods

### Overview

Composed of an interdisciplinary team and advisory board with experience in HCD, implementation science, psychosocial clinical interventions, and research methods and data, the Methods Core team supports one large hybrid effectiveness-implementation study (NIH R01), 3 exploratory pilot studies (NIH R34s; Multimedia Appendix 1), and at least 4 pilot projects (NIH R03s) during UWAC 2.0. These projects aim to improve clinical intervention and implementation strategy access and scale in diverse settings. The R01 Problem Solving Treatment-Aid (PST-Aid; NCT06494384; PIs: IB, PR, and SAM) will test a DDBT-designed decision support tool for Problem Solving Treatment (PST) in a large network of primary care clinics. R34 Research Units on Behavioral Intervention in Educational Settings (RUBIES; NCT06508515; PIs: KB and JL) will create a novel implementation strategy to support the delivery of evidence-based classroom supports for students with autism. R34 Trauma-Focused Cognitive Behavioral Therapy (TF-CBT; PIs: ARL and DW) will redesign and test an evidence-based clinical intervention for youth trauma for use in education settings. R34 Brief Intervention for School Clinicians (BRISC; PIs: EB, JVD, and Elizabeth McCauley) will adapt an existing set of effective implementation strategies to enhance delivery of a school-based engagement, assessment, brief mental health intervention, and triage strategy. Funding for all core projects began in 2023. Multimedia Appendix 2 outlines key details (eg, study design and sample) of each study. PST-Aid and RUBIES were part of UWAC 1.0 as R34 and R03 projects, respectively, highlighting how UWAC 2.0 activities build on previous accomplishments. UWAC funds pilot projects through a competitive solicitation process, with a particular focus on supporting and mentoring investigators from historically marginalized groups. All projects use the DDBT framework to address clinical intervention and implementation strategy usability, engagement, and appropriateness in partnership with local community collaborators. UWAC provides projects direct support for integrating methods and measurement approaches and professional development that centers diversity, equity, and inclusion (DEI) values.

### DDBT Constructs

Projects collect common data on DDBT mechanisms and constructs to determine the impact of modifying clinical intervention and implementation strategy targets. The Methods Core team maintains a list of recommended and required measures for projects to gather at each DDBT phase (“Center Measures and Guidance”; Multimedia Appendix 2). We developed Center Measures and Guidance to facilitate DDBT hypothesis testing and data management across UWAC, help teams select methods based on project design objectives, and satisfy NIMH reporting requirements. Center Measures and Guidance include 15 constructs with 26 quantitative and qualitative measures across DDBT phases. The Methods Core team provides project teams support with integrating and adapting these measures for projects through a consultation model. This is a shift from our approach in UWAC 1.0, where the Methods Core team provided more personalized measurement support to project teams. Multimedia Appendix 3 [83] outlines each construct with a description of related measures and activities, and relevance to each DDBT phase. The Methods Core team provides data management for all projects, maintains survey instruments in REDCap (Research Electronic Data Capture; Vanderbilt University), and conducts cross-project analyses. Each project is responsible for conducting its own analyses. The Methods Core team provides guidance on ensuring recruitment, data analysis, and dissemination practices incorporate diverse perspectives and accurately represent the lived experiences of participants to inform clinical intervention and implementation strategy redesign.

### DDBT Theory of Change Mechanisms

#### Usability

All projects are expected to report usability issues on existing or redesigned clinical interventions and implementation strategies and standardized usability metrics to the Methods Core team. Because interviews alone can be limiting for identifying usability issues because of issues with recall or challenges with describing behavior, UWAC projects are encouraged to combine interviews with other methods such as CWIS and TAP. This helps projects learn what a participant is considering doing next and why, better understand their in-the-moment goals, and identify misconceptions. For example, R34 BRISC will use TAP and a cognitive walk-through methodology with users to identify opportunities for redesign and improve implementation based on user needs [39]. The Methods Core team supports projects with adapting surveys (eg, SUS, IUS, and SUS) and implementing cognitive walkthroughs and usability testing for projects.

#### Engagement

We expect all projects to assess engagement quantitatively using the User Responsiveness Scale and qualitatively (ie, thematic findings from observation or other chosen methods) during the Discover and Test phases of existing and redesigned clinical intervention and implementation strategy. The Methods Core team developed the User Responsiveness Scale based on the Patient Responsiveness Scale [66]. The User Responsiveness Scale has 10 statements that cover participation and enthusiasm for a clinical intervention and implementation strategy that participants rate on a Likert scale. The original Patient Responsiveness Scale has demonstrated strong internal consistency (Cronbach α=0.86) and construct validity.

#### Appropriateness

R01 and R34s are expected to administer the IAM [79] or revised goodness-of-fit interview [84] during the Discover and Test phases of existing and redesigned clinical interventions and implementation strategies to probe areas of alignment and misalignment on goals and expectations, roles, etc. IAM is a 4-item survey and the leading instrument for measuring clinical intervention and implementation strategy contextual fit with good internal consistency (Cronbach α=0.87) and adequate test-retest reliability (*r*=0.73). The goodness-of-fit interview is particularly well-suited to probe on clinical intervention and implementation strategy appropriateness issues identified through IAM. We will use content analysis to analyze goodness-of-fit interview data. For example, R34 RUBIES will conduct goodness-of-fit interviews to explore the appropriateness of the RUBIES implementation strategy (“RUBIES-Team”) for the school environment using the CFIR domains to drive questioning.

#### Proximal Implementation Outcomes

##### Adoption and Reach

Adoption and reach are implementation outcomes specified in the CFIR [58] and Reach, Effectiveness, Adoption, Implementation, and Maintenance [85] frameworks. We expect projects to report on adoption and reach as part of the Design (if feasible) and Test phases. All projects will report adoption and reach of the intervention. For redesigned implementation strategies, the approach to measuring adoption and reach depends on the project. For example, R34 RUBIES defines adoption as educators’ first use of the RUBIES-Team at any point during the study and will measure reach for both RUBIES-Individual (ie, an original implementation strategy) or RUBIES-Team in three ways: (1) the number of other educators with whom trained educators share RUBIES strategies; (2) the number of other students with whom trained educators share RUBIES strategies; and (3) the number of other contexts and settings in which they applied RUBIES strategies.

##### Intervention and Implementation Strategy Fidelity

Fidelity is a core implementation outcome [58]. We expect projects to report on fidelity as part of the Design (for existing clinical interventions and implementation strategies) and Test phases. Teams choose an approach to measuring fidelity based on redesign goals. For example, R34 RUBIES rates paraeducator fidelity to treatment (eg, weekly ratings of the paraeducator’s homework completion and behavior support plan implementation). R34 TF-CBT will code session recordings using the Therapy Process Observational Coding Scale [86] and the Therapy Process Observational Coding Scale–Self-Reported Therapist Intervention Fidelity for Youth [87] at baseline, 3 months, and 6 months. Finally, R01 PST-Aid will measure initial and sustained fidelity using a PST Fidelity Scale. Initial fidelity will be measured as the number of sessions it takes providers to get certified; providers must have 2 sessions rated as “satisfactory” on the PST Fidelity Scale to receive certification. Sustained fidelity will be measured as the number of “satisfactory” sessions during the 6 months after certification, as measured by the PST Fidelity Scale.

##### Planned Adaptations

Characterizing adaptations, or redesign solutions, is key to all 3 Methods Core team aims to better understand and address challenges to clinical interventions and implementation strategies. We expect projects to characterize adaptations with FRAME or FRAME-IS [29] as part of the Design and Build phase, where planned and proactive changes will be made as a part of the redesign process. For example, R34 BRISC will analyze recorded intervention sessions using FRAME or FRAME-IS. The R01 PST-Aid will code randomly selected session audio recordings per client for fidelity and adaptations using FRAME or FRAME-IS. Projects will share adaptations made with the Methods Core team and describe whether these adaptions were made proactively (eg, as part of the redesign process in the design and build phases) or reactively (eg, unplanned or due to unanticipated obstacles in the test phase). Across projects, the Methods Core team will systematically categorize adaptations to examine solutions and the populations, organization types or structures, practitioner types, and clinical interventions and implementation strategies in which they work. Our objective is to identify transferable and unique solutions to different contexts and clinical interventions and implementation strategies. This information will ultimately inform an updated version of the library of redesign solutions.

##### Unplanned or Reactive Modifications

Reactive or unplanned modifications during the Test phase will be measured using a 17-item Center-developed measure based on the FRAME or FRAME-IS to assess the nature of modifications. Providers will self-report any changes they made while they administer the intervention.

#### Distal Service Recipient Outcomes

During the Test phase, projects are expected to collect clinical and functional outcomes. Teams will administer standardized assessments (eg, Patient Health Questionnaire [88], Quality of Life in Neurological Disorders [Neuro-QOL] [89], Satisfaction with Social Roles for adults [90], and the Neuro-QOL Social Relations Scale for youth [91]). The Neuro-QOL measures are widely used to assess functioning in usual social roles, activities, and responsibilities. The scales have been evaluated with thousands of participants from the general population of the United States and in clinical inpatient and outpatient settings who have a wide variety of presenting problems [89-91]. All projects will also use idiographic (ie, individualized) client outcome monitoring based on the Top Problems Assessment [92], an approach informed by goal attainment scaling [93,94] that has been found to be highly sensitive for monitoring clinical intervention outcomes and thus is preferred over standardized or nomothetic assessments by both practitioners and clients [95].

#### Demographic and Process Measures

##### Demographics

All projects are expected to collect participant demographic data mandated by the NIMH at all phases. Teams collect additional demographic data on the basis of the project needs. For example, the R34 RUBIES collects required educator and student demographics (eg, age, gender, and race) and additional data on school characteristics (eg, school size, percentage eligible for free or reduced lunch, racial and ethnic composition, percentage of English language learners, percentage in special education, annual funding for external resources, and per capita number of community-based organizations). Data will be tabulated to satisfy federal demographic and data reporting requirements as well as cross-project meta-analyses and comparisons.

##### User Needs and Experience

All projects are expected to clearly identify direct and indirect users and incorporate methods that address user needs. Explicit user identification produces more usable products and ensures that the design team does not underestimate user diversity [96] or create designs based on the team’s own needs [36,97,98]. clinical intervention and implementation strategy users should include the deliverers (eg, providers of clinical interventions) and recipients (eg, clients and implementation strategy targets such as administrators or clinicians). Identification of users for a clinical intervention and implementation strategy includes (1) generating a preliminary user list, (2) articulating the most relevant characteristics that reflect anticipated users, (3) describing and prioritizing main user groups, and (4) selecting typical and representative users [96]. For example, in R34 TF-CBT, direct users are school counselors and social workers who provide mental health services, as well as public school students with histories of traumatic stress. Potential indirect users in this project include caregivers of students. We included users who are diverse with respect to characteristics such as age (eg, students), race and ethnicity (eg, students and practitioners), culture (eg, students and practitioners), and clinical domain experience (eg, practitioners), which are features known to impact experiences of usability, engagement, or appropriateness [53,99-101].

Projects will use interviews to identify key challenges that users might face when applying clinical interventions and implementation strategies. Interviews consist of questions derived from HCD principles such as organizational and community culture, values, and challenges in applying clinical interventions and implementation strategies. For example, in R34 RUBIES, the team will interview educators about their existing opportunities to learn behavioral management strategies for students with autism who exhibit challenging behavior. Interviews will identify promising professional development approaches and areas to improve the existing RUBIES multifaceted implementation strategy. Additional interviews with school administrators or lead special educators are likely to surface critical organizational factors that can serve as design constraints for any subsequent redesign solution [102,103].

As described earlier, interviews can be supplemented through observation methods to better understand interactions in real-world settings. Projects can use an adapted form of TAP, where participants (eg, clinicians or clients) and researchers watch recordings of sessions while the participant explains what they were thinking in the moment. This approach can offer additional suggestions for improvement on the design of a clinical intervention or offer ideas for tools that could support the clinician during implementation. Interviews can also supplement comparative testing (eg, A/B testing) to explore and evaluate a broader landscape of design options and reach more robust solutions. A/B testing is an evaluation method in which ≥2 versions of a prototype are compared sequentially or in parallel to determine which version is easier to use and better meets user needs [104]. For example, R34 BRISC will build prototypes of digital asynchronous learning modules for novel users as well as posttraining support tools; initial prototypes will undergo comparative testing to finalize solutions to be evaluated in the Test phase. The pragmatic applicability and match of potential designs to the targeted service environments and resource constraints will be systematically addressed.

##### Participant Research Burden, Incentive Appropriateness, and Research Satisfaction

At the end of the Test phase, projects are expected to measure the burden of participation in the study. This instrument includes 6 questions to understand participants’ perceived burden of participating in the study, appropriateness of the level of compensation offered, and overall satisfaction with the study experience. This information will be used to help improve future protocols. Response frequencies will be tabulated for the 4 close-ended responses and themes will be summarized from open-ended responses.

##### Adherence to DDBT Process (DDBT Fidelity and Cost Measure)

All projects are expected to complete a Fidelity and Cost survey in REDCap about their application of HCD techniques at the end of each DDBT phase. We are developing the survey to systematically collect data on how the DDBT framework guides clinical intervention and implementation strategy redesign and link design activities with project goals. To facilitate teams in drawing on a range of methods, the Fidelity and Cost survey focuses on understanding which goals of each DDBT process teams completed (Figure 1) and the methods they used to support each goal. We will conduct descriptive statistical analyses and content analysis of data to understand the frequency of goals completed and HCD strategies used, links between strategies and goals, and modifications made to strategies during their use.

We will measure the costs of applying DDBT to help understand the resource requirements involved in its use, which can be a major challenge of HCD and coproduction methods [105]. Projects are expected to report total costs of redesign, reported through a Fidelity and Cost Survey in REDCap at the end of each phase (this is optional for R03 projects). We will aggregate activity-based costs (eg, time to create, complete, and analyze each activity; participant payments or time) across individuals, use budgets or other institutional records to assign hourly costs by role, and then add in any fixed costs (eg, materials and activity-specific software). We will calculate total DDBT costs, as well as phase-specific and activity-specific DDBT costs. Analyses will follow best practices by placing all US dollar values onto the same metric, including an index year to account for inflation; local or national average cost-of-living values to account for geographic variation in prices; and discounting of costs from different years due to preferences for delayed over immediate costs. We will conduct sensitivity analyses to examine the robustness of our cost estimates [106,107] by identifying areas of uncertainty in measuring units and prices for our ingredients, and then calculating costs across a range of plausible values (eg, we can substitute limits of 95% CIs for uncertain prices).

#### Team Collaboration, Trust, and Respect

At the end of each DDBT phase, R01 and R34s team members are expected to complete a survey that assesses satisfaction with the collaboration, impact of collaboration, trust, and respect. This survey is modeled after the Transdisciplinary Tobacco Use Research Center’s measure of Team Collaboration and Transdisciplinary Integration, which assesses satisfaction with the collaboration, impact of collaboration, trust, and respect [108]. Continuous review of outcomes will allow for critical assessment and course correction as needed and recommended by these bodies. Participation is confidential, and teams will receive an aggregate report of the number of team member participants and average scores for each item. Any free-response comments are additionally summarized. We will encourage teams to discuss results to improve their projects.

#### Community Participation in Research

The collaboration survey questions described earlier will be administered with additional questions to characterize the extent to which redesign teams engage users in a localized DDBT process. This instrument is based on an existing measure of community participation in research [109,110], which has been modified to target the design of clinical interventions and implementation strategies across 6 dimensions: identification of design issues, design activities, use of resources, design methods, indicators of success, and sustainability. Redesign teams will complete the measure at or near the end of each DDBT phase and then discuss ratings in an interview.

#### Investigator Satisfaction With the Support They Receive From the Center

At the conclusion of projects, we will ask investigators to share their level of satisfaction with support from UWAC through a 5-item Likert survey adapted from a survey used by the University of Washington IMPACT Center [111]. We intend to use these data to improve how the Methods Core team provides projects technical support.

### Data Analysis

The Methods Core team provides data management and guidance on all DDBT constructs. For Aim 3, fundamental comparisons are the differences in DDBT mechanisms (usability, engagement, and appropriateness) and implementation outcomes (adoption, reach, adaptation, and fidelity [and sustainment for the R01 PST]) for the original (unadapted) clinical intervention and implementation strategy versus the DDBT-informed (localized) clinical intervention and implementation strategy. We will conduct a (1) qualitative multiple case study analysis and (2) quantitative meta-analysis across projects. Case studies will examine each project’s context, implementation, mechanisms, and outcomes.

For each project, we will also develop analytical summaries to facilitate between-project comparisons. Using the constant comparative method [112], we will compare projects to group common and divergent themes. The meta-analytic synthesis will increase our inferential ability by combining results from the underpowered R34s. For the meta-analysis, each project’s mechanism and outcome will be summarized as a Cohen *d* effect size comparing localized DDBT with original clinical intervention and implementation strategy and corresponding 95% CI, using random effects weighting by the inverse of the within- and between-studies variance. Standard data screening and adjustments will be made to the data (eg, to limit the effect of outliers, they will be winsorized). Each project will be additionally advised on how to address possible confounders in analysis and reporting. For instance, projects will be encouraged to use naive participants in the Design and Build and Test phases, recognizing that adaptations of smaller elements of complex clinical interventions may require the participation of experienced participants during the Design and Build phase. In addition, randomization will occur at appropriate levels to avoid contamination by intralevel communication. Nonmonotonic missing data will be addressed via inverse probability weighting or multiple imputation, as appropriate [113]. The Methods Core will aggregate these data across projects into a series of working meta-analyses of the effectiveness of DDBT on each mechanism and outcome.

Finally, determining whether a DDBT-modified clinical intervention and implementation strategy leads to better implementation and clinical outcomes is ultimately a question of mediation. Although the initial R34 and R03 studies are not likely to yield large sufficient sample sizes to meaningfully test such an implementation mechanism question, the R01 PST will provide a direct test of the DDBT theory of change (Figure 2). Aggregating project data over time will allow us to eventually test a range of mediation-focused hypotheses via multivariate network meta-analyses.

### Incorporating DEI

During UWAC 2.0, we are improving the integration of DEI initiatives throughout Center activities. Projects selected for UWAC 2.0 and pilots must demonstrate potential substantial impact on clinical or public health outcomes, especially for historically marginalized communities. The Methods Core team provides mentorship on incorporating and adapting methods so that teams are positioned to conduct research that respects diverse populations and maximizes community benefits. Project teams will be provided training and consultation on the Adapting strategies to promote implementation reach and equity method, a 3-step process for adapting implementation science to promote equity, and expertise in methods for explicitly incorporating equity into the measurement of implementation outcomes [114]. We will also collaborate with projects to ensure diverse representation and decision-making during the DDBT phases, crucial stages where diverse viewpoints and demographically representative samples are essential. To facilitate diverse engagement, we will offer resources on building equitable research-practice partnerships, contextualizing implementation science to specific communities, and enhancing community collaborator capacities for community-engaged research. Consultation on quantitative critical research [115] will be provided to examine the treatment of race within quantitative methods and support equity testing through disaggregation, moderator exploration, and mixed methods triangulation.

UWAC additionally supports faculty and staff as part of its DEI work. The center team engages with historically marginalized investigators in planning and conducting center activities so that DEI efforts are integrated throughout center mentoring, pilot funding, methods support, and support for investigators planning future proposals. These measures include enhancing communication strategies based on team science [77] and avoiding a “minority tax,” which refers to assigning additional responsibilities to marginalized or underrepresented team members to promote diversity [116,117]. The Methods Core team also advises investigators on using patient-centered and nonstigmatizing language when reporting findings.

### Ethical Considerations

The University of Washington Institutional Review Board (IRB) approved materials and procedures for all 4 core projects and deemed projects minimal risk by June 2024 (PST-Aid: STUDY00017272; RUBIES: STUDY00017261; TF-CBT: STUDY00017262 and STUDY00019451; and BRISC: STUDY00017263 and STUDY00019682). All studies follow best practices across studies: review and collect informed consent and Health Insurance Portability and Accountability Act authorizations (when not waived) from participants; collect parental assent for individuals <18 years of age; compensate participants financially for their time and, when appropriate, with continuing education credits; and ensure participants are aware that they may opt out or leave the study at any time. When personal health information (eg, name, date of birth, and contact information) is collected, we preserve participant privacy and confidentiality by storing those identifiers separately from the study data and only linking them to study data via a code. The Methods Core team will provide support to pilot project teams on institutional review board applications after studies are funded.

## Results

### Overview

UWAC 2.0, including the 4 core projects detailed in Multimedia Appendix 3, received funding in June 2023. We provide a brief synopsis of each study’s progress as of January 2025 in the subsequent sections. Across studies, as well as the to-be-funded R03 pilots, we anticipate DDBT will result in changes to the clinical intervention and implementation strategy mechanisms, proximal implementation outcomes (ie, adoption, fidelity, reach, and adaptation), and clinical outcomes.

### R01 PST-Aid

This study began in the Design and Build phase, as the Discover phase was completed during an R34 in UWAC 1.0. For the Discover phase, the study team has completed a codesign workshop and user testing with patients and providers from a nonprofit, health informatics network with independent community-based health centers. The study is beginning the Test phase, and the team is recruiting the first cohort of providers. Providers will be randomized to receive training in either PST implementation as usual (ie, training and sessions supported by paper worksheets for practitioners and clients to use as they complete PST) or PST-Aid (ie, a web-based app that promotes practitioner-client collaboration in the use of PST for goal setting and action planning).

### R34 TF-CBT

At the time of submission, the study team has completed a task analysis of unadapted TF-CBT to prioritize components to develop scenarios for testing with students and school-based mental health practitioners. The study has finalized TF-CBT user-testing scenarios and are actively recruiting students and practitioners to begin the first of 3 waves of testing for the study. These data will then inform the redesign of TF-CBT, which aims to be more usable in school settings.

### R34 RUBIES

Discover and Design and Build phase study activities, including focus groups, cognitive walkthroughs, and user testing with paraeducators and other school personnel (eg, principals and teachers), are complete. These data are being used to inform the Test phase where the study team is recruiting paraeducators and students and their caregivers to begin RUBIES training. Once recruited, this Test phase will include a 2-year randomized controlled trial enrolling paraeducators and students who will then be randomized to 1 of 2 implementation strategies: RUBIES-Individual or RUBIES-Team.

### R34 BRISC

This study is scheduled to begin in April 2025, and the study team has received institutional review board approval.

## Discussion

### Charting New Research Directions

Our vision is to address persistent issues with usability, engagement, and appropriateness that are barriers to clinical intervention and implementation strategy use by drawing from the fields of HCD and implementation science. The first iteration of the center advanced our understanding of how DDBT can guide clinical intervention and implementation strategy adaptations for uptake in historically marginalized communities. During UWAC 2.0, we aim to continue serving as a multidisciplinary incubator to find viable solutions for improving implementation of clinical interventions and implementation strategies using DDBT through the R01, R34s, and pilot projects. Table 1 summarizes potential outputs and future directions by aim. Focusing on usability, engagement, and appropriateness and providing and testing ways to measure engagement in a clinical intervention and implementation strategy context is particularly novel. UWAC 2.0 will further test the robustness of the DDBT theory of change, expand the potential evidence base for its utility in combining HCD and implementation science for clinical intervention and implementation strategy redesign, and add to the field’s understanding of how to apply DDBT to a variety of clinical intervention and implementation strategies and contexts.

Our experiences underscore a benefit of developing additional resources for DDBT, HCD, and implementation science. UWAC 1.0 outputs contributed to foundational conversations on the intersection of HCD and implementation science and produced potential pathways to address conceptual overlap and distinctions [43] and terminology [42]. We additionally developed resources such as CWIS [39], IUS [37], the ISUS [39], and a typology of modification targets and usability issues [53]. We have accumulated substantial experience adapting common HCD methods for different contexts and communities, and we hope these adaptations can support future teams in their use these methods. We plan to build on these methodological advancements, which have been used beyond the UWAC team by a broader research community interested in HCD and implementation science methods and measurement. As we learn with UWAC 2.0 project teams, we will continue to identify and develop additional resources for UWAC project teams and the broader research community on specific methods, team science, equity-oriented design practices, and grant writing. Developing additional resources aligns with the Methods Core team shifting to a consultation model on using the DDBT framework during UWAC 2.0. UWAC projects receive technical support from the Methods Core team with greater emphasis on building local capacity to apply DDBT rather than having the Methods Core team members conduct some of the DDBT activities.

**Table 1:** Expected center-level outputs and future directions by aim.

Aim	Expected outputs	Potential future work
Aim 1: identify clinical intervention and implementation strategy modification targets to improve usability, engagement, and appropriateness in accessible nonspecialty settings (Discover phase)	Updated Typology of Modification Targets that expands the previously identified usability issue categories [53] New insights on engagement and appropriateness issues in clinical intervention and implementation strategy redesign	Incorporate additional findings from non–UWAC^a^-funded projects that have used the typology of modification targets
Aim 2: develop redesign solutions with local teams to address clinical intervention and implementation strategy modification targets (Design and Build phase)	Updated Library of Redesign Solutions	Recommendations on how to approach measuring engagement and appropriateness with a focus on clinical intervention and implementation strategy redesign
Aim 3: determine if redesign affects changes in usability, engagement, and appropriateness (Test phase)	Integrated cross-project analyses to demonstrate the impact of DDBT^b^-informed redesign	Further expansion of DDBT and associated methods to new domains in health and social services

^a^UWAC: University of Washington Advanced Laboratories for Accelerating the Reach and Impact of Treatments for Youth and Adults with Mental Illness Center.

^b^DDBT: Discover, Design and Build, and Test.

### Limitations

UWAC 2.0 and the DDBT framework represent a robust effort to integrate HCD with implementation science, although several methodological limitations merit consideration. The standardized measures we are using to assess usability, appropriateness, and engagement have evidence of psychometric soundness, but these measures have not yet been evaluated in all the contexts in which they will be applied. Redesign solutions developed by local teams may rely primarily on context-specific adaptations, which may not generalize to other settings and may not address systemic barriers that impact usability or accessibility. Therefore, reactive modifications (ie, primary outcome) needed to address additional barriers may not be reduced. In this work, there is a tension between our desire to standardize DDBT phases and measures, to facilitate center-wide learning, with promoting the adaptability and flexibility to adapt to specific goals, contexts, and populations that is necessary for good design projects. This, combined with the variability of research project contexts, the small sample sizes of redesign teams and subjective nature of the proposed mechanisms may impede our ability to make inferences across projects.

### Conclusions and Impact

There is a pressing need to ensure that clinical interventions and implementation strategies are easily implementable and meet the needs of the communities they aim to help. Integrating HCD and implementation science offers promising approaches to tackle this challenge. UWAC 2.0 expands and strengthens our efforts to ensure that accessible community service settings and marginalized communities see the benefit of decades of research on effective clinical interventions and implementation strategies.

## Appendix

Multimedia Appendix 1 Peer-review report by the National Institute of Mental Health Special Emphasis Panel - National Institute of Mental Health - Advanced Laboratories for Accelerating the Reach and Impact of Treatments for Youth and Adults with Mental Illness (National Institutes of Health, USA).

Multimedia Appendix 2 Summary of projects.

Multimedia Appendix 3 Center measures and guidance.

## Abbreviations

BRISC: Brief Intervention for School Clinicians
CFIR: Consolidated Framework for Implementation Research
CWIS: Cognitive Walkthrough for Implementation Strategies
DDBT: Discover, Design and Build, and Test
DEI: Diversity, Equity, and Inclusion
FRAME: Framework for Modifications and Adaptations of Evidence-Based Interventions
FRAME-IS: Framework for Modifications and Adaptations of Evidence-Based Interventions for Implementation Strategies
HCD: human-centered design
HCI: human-computer interaction
IAM: Intervention Appropriateness Measure
ISUS: Implementation Strategy Usability Scale
IUS: Intervention Usability Scale
Neuro-QOL: Quality of Life in Neurological Disorders
NIH: National Institutes of Health
NIMH: National Institute of Mental Health
PI: principal investigator
PST: Problem Solving Treatment
PST-Aid: Problem Solving Treatment-Aid
REDCap: Research Electronic Data Capture
RUBIES: Research Units on Behavioral Intervention in Educational Settings
SUS: System Usability Scale
TAP: think-aloud protocol
TF-CBT: Trauma-Focused Cognitive Behavioral Therapy
UWAC: University of Washington Advanced Laboratories for Accelerating the Reach and Impact of Treatments for Youth and Adults with Mental Illness Center

## Data Availability

Clinical outcome data for the R01s and R34s will be made publicly available through the National Institute of Mental Health National Data Archive. The quantitative datasets generated and analyzed during this study are available from the corresponding author on reasonable request.
