# Supplementary material for: Harnessing Human-Centered Design for Evidence-Based Psychosocial Interventions and Implementation Strategies in Community Settings: Protocol for Redesign to Improve Usability, Engagement, and Appropriateness
Source: JMIR Res Protoc. 2025 Jan 29;14:e65446. doi: 10.2196/65446 (PMC11822321; doi:10.2196/65446)
Supplement: Multimedia Appendix 1 [file resprot_v14i1e65446_app1.pdf]

2P50MH115837-05 LYON, AARON

RESUME AND SUMMARY OF DISCUSSION: This competitive renewal, the UW Alacrity Center for Psychosocial Interventions Research, will focus on the implementation of evidence-based psychosocial clinical interventions (CI) in primary care clinics and schools. The signature project will test a Discover, Design/Build, Test (DDBT) tool for Problem Solving Treatment in primary care clinics. The exploratory projects involve using implementation strategies (IS) to redesign an intervention for autism spectrum that will be delivered in classrooms, modifying CI for youth trauma that will be delivered in education settings, and improving the delivery of school mental health assessments and triage through IS. The Center was productive in its previous funding cycle, which resulted in 23 project awards and 35 publications. A website and educational materials were also developed. The Administrative Core (AC) will manage the Science and Policy Board, Stakeholder Advisory Board, pilot projects, mentorship of early-stage investigators, dissemination, and the evaluation of the Center. The collaboration between this Center and other Advanced Laboratories for Accelerating the Reach and Impact of Treatments for Youth and Adults with Mental Illness (ALACRITY) Centers was impressive. Concerns were raised regarding a lack of clarity with the training hub and the evaluation metrics. The Methods Core (MC) will provide support to and synthesize results from the Center's projects. The MC is a notable strength of the Center. It was noted; however, that the level of support that the MC will be able to provide to Center investigators is not clear. The Center has a number of strengths. The investigators are excellent. Drs. Lyon and Munson are well-qualified to serve as Co-Directors of the Center and they will be supported by a strong team of investigators. However, it was noted that the Co-Directors appear to be heavily involved in multiple components of the Center, which might be overly taxing. It was also not clear if there was sufficient biostatistical support in the Center. The environment is also excellent. The University of Washington (UW) will offer ample infrastructure and resources to complete the proposed work. The involvement of the Department of Human Centered Design and Engineering was a notable strength. If successful, the Center has the potential to improve the care that is provided in primary care and schools. A concern raised by most reviewers was the lack of rationale provided for the shift from adults in primary to the heavy focus on school based mental health. All of the exploratory projects are focused on care that is delivered in schools while the signature project is focused on primary care. There appeared to be a lack of integration, cohesion, and synergy across the studies. Reviewers also raised concerns with the innovation of the Center, which seemed to be an incremental change from the previous funding cycle. The approach was generally strong and appeared to target key issues of IS, but several weaknesses were noted. The DDBT paradigm did not appear to fit as well with some of the projects. It appears that most of the sample is anticipated to be white, which will not allow for separate statistical testing to be completed on race. There might have been additional attention given to context and culture in the Center. There was some unclarity regarding some of the statistical analysis plans and power analysis. Overall, this was a very good Center, but some of the noted weaknesses slightly detracted from the potential impact of this work on the field of CI in primary care and schools.

DESCRIPTION (provided by applicant): The purpose of the UW ALACRITY Center is to address critical problems in the implementation of evidence-based psychosocial clinical interventions (CI) (e.g., psychotherapies) in nonspecialty service settings that are accessible to underserved communities (i.e., primary care clinics and schools). Use of CI is inhibited by longstanding problems with the usability, contextual fit, and engagement of both those CI and the implementation strategies (IS) (e.g., consultation models) the support them. This situation also results in high rates of "reactive adaptations" of CI/IS by their intended users in many settings where they are deployed. Building on our learnings and successes over the past 4 years, our interdisciplinary research team will draw from the fields of human- centered design (Drs. Munson, Fogarty), implementation science (Drs. Lyon, Bennett, Locke, Fortney, Bruns, van Draanen), psychosocial CIs (Drs. Areán, Raue, McCauley, Bearss, Walker), and research methods/data analysis (Drs. Pullmann, Dopp). The Center represents a unique partnership between the School of Medicine's Departments of Psychiatry and Family Medicine, the Department of Human-Centered Design and Engineering, the Department of Computer Science, the School of Social

Work, the School of Public Health, and the College of Education. The Center also bridges UW's many resources: The Institute for Translational Health Sciences (the UW CTSA), the AIMS Center (UW implementation center for collaborative care), the SMART Center (UW implementation center for education sector services), the WWAMI-region Practice Research Network (WPRN, primary care practices in Washington, Wyoming, Alaska, Montana and Idaho), and the MHTTC Northwest School Mental Health Network (public school districts across Washington, Oregon, Alaska, Idaho). The Administrative Core (AC) will serve as the communication hub between Center cores and advisory boards; oversee the selection of pilot studies; and coordinate mentoring, evaluation, and dissemination. The Methods Core (MC) will provide research infrastructure to projects and support use of our Discover, Design/Build, Test (DDBT) framework to address CI/IS usability, engagement, and appropriateness in partnership with local stakeholders. The MC will also compile project data to update and refine our Typology of CI/IS Modification Targets and Library of Redesign Solutions for dissemination and assess costs of DDBT-driven redesign. Research projects will collect common outcome data to determine the impact of modifying CI/IS targets. The Signature R01 will test a DDBT-designed decision support tool for Problem Solving Treatment in a large network of primary care clinics. R34 #1 will create a novel IS to support delivery of evidence-based classroom supports for students on the autism spectrum. R34 #2 will modify a well-researched CI for youth trauma for use in education settings. R34 #3 will adapt an existing effective IS to enhance delivery of school mental health assessment and triage. All four projects, and future pilot projects, will test effects of redesign on usability, appropriateness, engagement, implementation outcomes, and patient outcomes.

PUBLIC HEALTH RELEVANCE: Psychosocial clinical interventions are the preferred mode of treatment for most people seeking care for mental health problems – particularly among people of color and those living in poverty in both rural and urban communities – but very few people have access to them because of the problems with usability, contextual fit, and engagement experienced when those interventions and the implementation strategies designed to support their use are deployed in accessible settings like primary care medicine and schools. The UW ALACRITY Center will address these issues by advancing its novel framework for redesigning clinical interventions and implementation strategies, drawing from the fields of human-centered design and implementation science. Across projects, the UW ALACRITY team will work with local practitioners and other stakeholders to develop and test four new solutions for addressing problems in the usability, contextual fit, and engagement of clinical interventions and implementation strategies in primary care and schools and will support additional pilot studies over the 5-year timeline.

CRITIQUE 1

Significance: 2
Investigator(s): 1
Innovation: 3
Approach: 1
Environment: 1

Overall Impact:

This is a competing renewal application for an Advanced Laboratories for Accelerating the Reach and Impact of Treatments for Youth and Adults with Mental Illness (ALACRITY) Center which has shown excellent productivity, and has increasingly become a national resource for training in their Discover, Design/Build, and Test framework (DDBT).

The leadership and investigators covering the broad range of skills necessary.

The renewal addresses the problem of implementation of clinical interventions in the settings where they are deployed, particularly in communities of color and rural populations. The DDBT design methodology was developed during the past four years as part of the current ALACRITY Center and it

has already achieved wide acceptance beyond the University of Washington (UW). This renewal uses the results of the first Center to develop a “typology of targets” and a “library of exemplary solutions” to help focus future DDBT-guided design efforts.

The 3 multidisciplinary R34 projects are relatively similar projects designed to apply this methodology to current significant clinical problems: a school-based behavioral skills intervention for autism, Trauma-Focused Cognitive Behavioral Therapy (TF-CBT) redesign for delivery in schools for youth with posttraumatic stress disorder (PTSD), and a 4-session school-clinician based intervention broadly targeting internalizing and externalizing disorders and other academic, peer or family issues. The R01 project is designed as a test of DDBT-designed decision support tool to improve a Problem-Solving Treatment in Community Health Centers.

All research projects obtain qualitative and quantitative data on usability, engagement, implementation outcomes, and client-reported outcomes.

The methodology and focus on implementation science well fit the goals of ALACRITY Centers. The specific targets in the four projects in this renewal are all addressing high value goals. The work appears entirely feasible. Further experience with and dissemination of the DDBT design methodology has potential significant impact for work by this group and others.

1. Significance:
Strengths

- The renewal addresses how to improve implementation of evidence-based psychosocial clinical interventions in non-specialty service settings, including schools (in the 3 exploratory project studies) and in primary care clinics (the Signature project study). Given scarcity of mental health care delivered by specialists, especially but not exclusively in disadvantaged groups, this is an enormously important question.
- The exploratory studies, DDBT-modified versions of existing treatments with evidence of efficacy that are great targets for school-based treatment improvements, are promising, of reasonable size to show feasibility, and may provide a meaningful test of non-inferiority (though the confidence limits will be relatively wide).
- Important psychiatric problems are targeted in the exploratory studies (autism, depression, and mixed psychiatric disorders mainly though not exclusively internalizing disorders).
- The methodologies proposed in the R34 studies and R01 study are very well chosen. The investigator teams have, in aggregate, all skills necessary to complete the work, and there is good documentation of access to and prior success in recruiting these populations.
- The current ALACRITY Center that this work builds on has shown significant synergy that this group of prominent investigators have obtained by their collaboration, strongly supporting the value of the Center.
- The Center explicitly focuses on people of color and both urban and rural communities.
- The number of informants necessary to detect at least 80% of usability problems is asserted to be 10±2 subjects and this is supported by a reference. However, that assumes that the sample is representative of the entire population. In several of the projects a significantly larger testing cohort is selected to ensure input from different perspectives.

Weaknesses

- While the proposed Signature study is interesting and extremely well designed, the prior work is not yet published, appears to be on a small number of subjects, and is only partially described.

2. Investigator(s):
Strengths

- Excellent combination of experienced investigators and junior faculty across Cores and projects.
- Investigators from fields of human-centered design, implementation science, psychosocial treatments, research methods, and data analysis. Draws faculty from psychiatry, family medicine, department of human-centered design and engineering, computer science, social work, public health, and education.

- Overall Center co-Directors, Drs. Aaron Lyon (Psychiatry and Behavioral Sciences) and Sean Munson (Dept of Human Centered Design & Engineering) have led large studies, have collaborated with each other and many of the other investigators, and have all necessary skills to successfully lead this complex Center.
- The leadership and investigators of the Methods Core and the four studies are well chosen and have all needed expertise.

Weaknesses
- None noted by reviewer.

3. Innovation:
Strengths
- The further improvement of the Discover, Design/Build, and Test framework (DDBT) and the inclusion of the “typology of targets” and a “library of exemplary solutions” developed in the current ALACRITY Center are highly innovative.

Weaknesses
- None noted by reviewer.

4. Approach:
Strengths
- The overall approach proposed in this Center is rigorous, targets key issues of implementation science for dissemination of treatments into schools.
- The approaches used are robust and unbiased.
- For all studies subject availability, recruitment, retention plans are good.
- Incorporation of stakeholders into all projects.
- Prior work in this Center and plans all show extremely effective sharing of research resources to other research groups including other ALACRITY Centers.
- Data analytic plans are state-of-the-art, comprehensive, and entirely appropriate for the proposed work.

Weaknesses
- None noted by reviewer.

5. Environment:
Strengths
- Institutional support, computers and other equipment, and patient populations are all sufficient for the proposed studies.
- Administrative and data resources are sufficient.

Weaknesses
- None noted by reviewer.

Milestones:
Comments:
- Milestones are clearly present.

Administrative Core
Strengths
- The Core leaders, Drs. Lyon and Munson, co-direct the Administrative Core.
- The first primary aim, facilitate use of DDBT in improving clinical interventions (CI)/ implementation strategies (IS) in primary care and school settings, builds on the success of the current ALACRITY Center. The excellent record doing exactly this suggest continued success.
- The Science and Policy Board has expertise in intervention adaptation, implementation outcomes, implementation in education, poverty/inequality, statistics, mental health services,

mental health (MH) in primary care settings and schools, and human-computer interaction. The needed skills are well represented.

- The Stakeholder Advisory Board is from primary care medicine in underserved communities, school-based MH stakeholders, and representatives from the advisory board for the R01 and R34 projects. The project-specific board also include stakeholders.
- The pilot project funds will be enhanced by a commitment of UW to dedicate substantial funds to hire more faculty from historically marginalized groups. The Center will partner with other organizations in Seattle and across the five-state region to advertise and recruit pilot study research. The plans appear appropriate.
- The request for applications (RFA) for pilot studies will be developed with input from the Stakeholder Advisory Board. The Center will fund up to 6 projects of 1 year duration. The inclusion of input from the Stakeholder Advisory Board is great.
- Structured mentoring will be provided for early-stage investigators and from historically marginalized groups. Clear details are provided and the success of the current ALACRITY Center in mentoring and promoting early-stage investigators is impressive.
- A "Grant Writers' Boot Camp" is provided for those about ready to submit early-career K awards (three times per year).
- The Center will continue and expand the Website and provide additional training content and resources.

Weaknesses

- None noted by reviewer.

Methods Core

Strengths

- Methods strongly use prior and ongoing input from researchers, stakeholders, and experts in other fields.
- Considerable emphasis on exporting DDBT in general and especially as applied to clinical interventions and implementation strategies.
  - DDBT includes identification of needs, contextual observation of users in their settings, comparative testing, mixed-methods assessment of mechanisms.
- Use of typology of modification targets based on results of prior Center to add "usability", "appropriateness" and "engagement" proposed mechanisms.
- Developed a library of 12 potential redesign solutions from current Center work.
- Examination of factors at multiple system levels (organizational setting, individuals, intervention).
- Clear elucidation of approach to select particularly impactful modifications in CI/IS capacity.
- DDBT incubator providing support and guidance across DDBT efforts appears quite successful.
- Good application of contemporary digital tools, e.g., Slack, to facilitate iterative design.
- Very broad span of skills from the directors and Co-Is.
- Inclusion of contextual observation of users in their settings.
- Excellent inclusion of diversity, equity, and inclusion (DEI) issues including getting diverse viewpoints on design issues and solutions and during test phase.
- Analytic approaches to all three Aims are appropriate and detailed.
- Appropriate and detailed dissemination plans.
- Using REDCap for data portal and repository.
- Plans to make all products available to researchers outside the Center.

Weaknesses

- None noted by reviewer.

Research Projects

Strengths

- Exploratory Project (EP) 1: Goal is to develop implementation strategies for behavioral-skills interventions for autistic youth to be provided by lay providers in public schools using DDBT framework.
- EP1: Tests mechanisms of adoption, fidelity, adaptation, and reach. Will capture costs of redesign using DDBT methods.
- EP2: Goal is to deliver TF-CBT in schools addressing usability limitations using DDBT paradigm. Original TF-CBT will be compared to redesigned TF-CBT in sample of 24 school clinicians and 48 students. Randomization is done on school clinicians (each of whom will treat 2 students).
- EP2: will use 15 practitioners and 15 students during testing group.
- EP3: will use 10 school counselors and 5 administrators for design process. Aim 2-3 study will include 12 school clinicians and 60 students. School districts divided into half lower-resource rural districts (N=2) and half higher-resource suburban districts (N=2). Half of districts will get original school intervention and half the improved intervention.
- Signature Project (SP): goal is to develop a web-based automated decision support tool supporting Problem Solving Treatment (PST). Test of new treatment will include implementation outcomes (fidelity, adoption, reach, and reduced adaptations) and changes in usability, engagement, and appropriateness, and reduction in client depressive symptoms and improved functioning.
- SP: 60 practitioners from 20-30 schools will be trained and then practitioners will be randomly assigned to two treatments. Each practitioner will treat on average 5-6 subjects. Randomization is at level of practitioner with stratification by rural/urban clinics and practitioner median years of experience.
- SP: Use of the OCHIN.
- SP: Tests of mediation will be made.
- SP: Power to detect a non-inferiority margin of d>0.29.

Weaknesses

- EP1: Not clear why 85% female rate is expected. Rate of expected non-white subjects is about 54% but expected to be 16% African American (AA), 12% Hispanic, 16% Asian and 10% other so won't have sufficient subjects to look and specific race/ethnicity differences.
- EP2: it is anticipated that 2/3 of subjects will be white.
- SP: PST-Aid prototype was piloted; however, information does not appear to be provided on the number of practitioners who piloted it. It is reported that 3 clients using PST-Aid reported improvement on the Patient Health Questionniare-9 (PHQ-9) (delta of 9 points) and 3 clients on PST as usual reported improvement (delta of 4.7). It is not clear that amount of pilot work robustly supports this R01-size study as the next step in examining this potentially significant improvement in PST.
- SP: 2/3 of subjects will be white. Approximately 21% will be black (so not really sufficient for separate statistical testing.

Study Timeline - Overall

Comments:

- Timeline for each of the projects is clearly detailed and appropriate.

Protections for Human Subjects

Acceptable Risks and/or Adequate Protections

- There are no human use concerns or comments.

Data and Safety Monitoring Plan (Applicable for Clinical Trials Only):

- Acceptable
    - Appropriate data safety and monitoring plans presented.

Inclusion Plans

- Sex/Gender: Distribution justified scientifically.
- Race/Ethnicity: Distribution justified scientifically.
- For NIH-Defined Phase III trials, Plans for valid design and analysis: Not applicable.
- Inclusion/Exclusion Based on Age: Distribution justified scientifically.

Vertebrate Animals
Not Applicable (No Vertebrate Animals)

Biohazards
Not Applicable (No Biohazards)

Renewal
- A strong renewal application building on prior work over the past four years.
- As outlined in the progress report, the University of Washington ALACRITY Center (UWAC) 1.0 support results in 23 grant awards and 35 publications. It supported 3 collaborations with other ALACRITY Centers. UW teams had 10 grant submissions with 6 funded so far. Other metrics covering supporting early-stage investigators is similarly impressive.
- UWAC 1.0 supported the development of a website with 16 educational videos as well as measures and methods.

Applications from Foreign Organizations
Not Applicable (No Foreign Organizations)

Select Agents
Not Applicable (No Select Agents)

Resource Sharing Plans
Acceptable
- Resource sharing plans presented are appropriate and conform with NIH requirements.

Authentication of Key Biological and/or Chemical Resources
Not Applicable (No Relevant Resources)

Budget and Period of Support
Recommend as Requested

CRITIQUE 2

Significance: 2
Investigator(s): 2
Innovation: 3
Approach: 3
Environment: 1

Overall Impact:
The overall goal of this P50 renewal is to improve the implementation of clinical interventions (e.g., psychotherapy, counseling, case management) for underserved populations (i.e., people of color, living in poverty) seeking care in safety-net primary care and school settings. There is also a focus on internalizing and externalizing conditions (e.g., depression, anxiety, disruptive behavior). This is a competitive renewal application (University of Washington ALACRITY Center [UWAC] 1.0); the prior application had a similar focus and developed a strategy called Design/Build and Test framework

[DDBT] in similar settings. The Administrative Core focuses on supporting the R03 pilot projects, Team Science, and Grant Writer Bootcamps, and Center evaluation and research dissemination. The Methods Core focuses on use of DDBT methods, building on a typology and library of learnings from UWAC 1.0, and examining the effectiveness of these methods. The project's R01 tests a DDBT-designed decision support tool for problem-solving treatment in a network of 1,000 safety net clinics. The 3 R34s focus on: Behavioral intervention in Schools (Research Units for Behavioral Intervention in Schools [RUBIES]), Trauma-focused Cognitive Behavioral Therapy (TF-CBT), and Brief Interventions for School Clinicians (BRISC).

Overall, this is a strong renewal, which has minor to moderate weaknesses mainly in some of the specific research projects proposed. The overall project plan and the Methods Core are particular strengths; they have learned a lot in UWAC 1.0 in terms of both clinical intervention (CI)/implementation strategy (IS) methods as well as the best ways to run the Center as a whole. The Center has been productive in terms of its pilot research program (many R03s converted into funded grants) and has a strong publication record. The methodological similarities across projects as well as a shared conceptual model are significant strengths, which will allow each project to learn from another. Also many logical meta-projects have been proposed based on findings from the Center as a whole. The Center has been collaborative with other Advanced Laboratories for Accelerating the Reach and Impact of Treatments for Youth and Adults with Mental Illness (ALACRITY) Centers and has a strong commitment to equity and inclusion.

The most significant weakness of this renewal is the fit of some of the component projects with the overall Center paradigm. The DDBT framework seemed to work best with the R01 and R34 - P002, in which there was an intervention that clearly needed redesign to a new context. It did not work so well for R34-P001 and R34-P003, because in the first case it seemed like the intervention had already undergone considerable redesign, and in the second case, the intervention was actually designed for the setting that it was being redesigned for. Other moderate weaknesses included a heavy emphasis on school health (versus primary care) in both the study team and the projects, and a general lack of emphasis on innovation. The project as a whole seems like an incremental change to UWAC 1.0, which was successful, and not really a reimagining of what the Center could be.

1. Significance:
Strengths

- School and primary care settings have poor access to evidence-based CIs, and when they do have access to them, they are often implemented with poor fidelity due to poor fit with their setting.
- The project's focus on internalizing and externalizing conditions makes sense for schools and primary care, corresponds well with the Research Domain Criteria (RDoC), and is particularly timely given mental health consequences of Covid-19. It also corresponds with NIMH's Notice of Special Interest (NOSI) regarding mental health interventions in school settings.
- The DDBT framework, which was used in UWAC 1.0, is a strong framework for adapting evidence-based CI's. In this renewal the DDBT teams will be locally-led with cross-site collaboration as opposed to centrally led. The focus is on usability, appropriateness, and user engagement. This is an expansion from the prior focus which was primarily on usability. In addition, from a methodological standpoint, they are focusing on creating and refining a library of CI/IS modification targets and redesign solutions matched to these targets, which are meant to be generalizable.
- The overall Center has been productive in terms of publications. They have had a good success rate in getting their R03s funded as R34s (6/8 funded as R34s and one with fundable score). Also it is commendable that these projects completed data collection considering the Covid pandemic.

Weaknesses

- It could have been better articulated how UWAC 2.0 will be different than UWAC 1.0. It seems like the changes are rather minor overall. A table might have been helpful.

- The renewal is about school and primary care settings. The R01 is about primary care settings. However, none of the R34s are about primary care settings. It does seem like there is a greater focus on school health than primary care.

2. Investigator(s):
Strengths
- Center directors: Dr. Lyon is a clinical child psychologist whose research focuses on increasing accessibility and effectiveness of psychosocial interventions in school settings, with a strong focus on human-centered design. He has been the PI of an NIIMH R01 focusing on trauma-focused CBT implementation in schools, as well as an R34 and several recent R21s focusing on implementation science in schools. He also has been the PI of multiple Institution of Education Sciences (IES) grants. He previously directed the UWAC 1.0 Center for part of its funding period. He was promoted to full professor at UW last year. Thus, he has the requisite experience to lead this effort. Dr. Munson is an associate professor of Human Centered Design (HCD) and Engineering at UW. It seems he is a new investigator per NIH rules, and he does not have any R01 grants or R01 equivalents as PI, although he does have an IIS PI grant, and was the PI of one of the prior ALACRITY R34s. He has a strong mentorship record of trainees at multiple career stages and he has expertise in HCD and informatics which will be helpful. Both investigators previously co-directed the Methods Core of UWAC 1.0, so they have a history of collaboration, which is a strength. Having one clinician and one non-clinician is a strength.

Weaknesses
- This is the largest such project that Dr. Munson has directed and he might not really have the needed experience as an NIH PI. However, it is an MPI situation and Dr. Lyon has this experience. Minor weakness.
- Drs. Munson and Lyon are Center directors, Administrative Core (AC) co-directors, and Methods Core (MC) co-directors. This seems like a lot. It also seems like it would be more optimal if the AC and MC were a little more independent (minor/moderate).
- Overall, the key personnel lean heavily toward psychology. It is somewhat telling that a Center that is supposed to be “about primary care” only has one primary care provider as key personnel. There just seems to be more focus on school settings than primary care settings. It seems that primary care was more a focus of UWAC-1, so perhaps they have some prior expertise. Minor/moderate weakness.

3. Innovation:
Strengths
- Their multidisciplinary approach to addressing problems in CI/IS for mental health in community settings, addresses a high priority and an under-researched area.

Weaknesses
- It seems that this renewal is an incremental change from UWAC 1.0 instead of an innovative new look at the problems it seeks to solve. Maybe that’s okay since the Center addresses a highly significant problem type, and the Center has been productive. However, it seems like there was a missed opportunity here to take some creative new approaches (minor).
- The innovation sections of the research projects were rather short. It just didn’t seem like a high priority was placed on innovation here (minor/moderate). It mainly seems like they are continuing the successful process/approach that they had with UWAC 1.0.

4. Approach:
Strengths
- Using similar outcomes and conceptual models in all of the projects is a significant strength and fits well with the “whole is more than sum of parts” aspect of ALACRITY. A lot will be learned across projects.

- Likewise there were some nice methodological similarities between the component projects in terms of user testing and pilot trial design and analysis. That aspect of this research is really appreciated; there will be a lot of synergy and teams can learn from one another.
- There is also a good economy of scale and expertise possible here, in which individual projects can draw on shared administrative and methods resources, which is one of the benefits of having a Center.
- The team seems to have all of the community partnerships and experience working within school districts to facilitate recruitment. For the Signature Project (SP), the partnership with OCHIN gives them access to a high-risk patient population distributed over many clinics. They had really substantive stakeholder engagement in UWAC 1.0, which is a strength.
- Recruitment overall seems feasible.
- The Cores will monitor the overall efficacy of each project and the Center as a whole. Those plans are well-detailed.

Weaknesses

- The “fit” of the DDBT paradigm to each of the component projects was variable. It seemed to work best with the R01 and R34 - P002, in which there was an intervention that clearly needed redesign to a new context. It did not work so well for R34-P001 and R34-P003, because in the first case it seemed like the intervention had already undergone considerable redesign, and in the second case, the intervention was actually designed for the setting that it was being redesigned for. Moderate weakness overall.
- As a general comment, the Center overall approach and Cores were very strong, and its component projects (especially some of them) were not as strong. See specific project comments for detail. This brought down the overall approach score.
- None of the R34s from UWAC 1.0 converted to R01s. However, one did result in a comparably-sized foundation grant. It does seem like they are providing good resources to early career people, however.

5. Environment:
Strengths

- The environment at University of Washington is more than adequate, and the success of the prior UWAC 1.0 shows that they have the resources they need to be successful.
- UW has a department of Human Centered Design and Engineering, which is a unique resource.
- There are several other university-wide centers related to mental health implementation (School Mental Health, Assessment, Research, and Training [SMART] and Advancing Integrated Mental Health Solutions [AIMS]) which will also complement resources. UW’s Clinical Translational Science Award (CTSA) will also support training and provide resources.

Weaknesses

- None noted by reviewer.

Milestones:
Comments:

- Center-wide milestones are provided in the Methods Core (MC) and seem reasonable.

Administrative Core - 1

Strengths

- Drs. Lyon and Munson, center co-directors, are directors of the AC. They are certainly qualified to do so; however, it is worth noting that have a lot of responsibilities in this project.
- Dr. Areán (former UWAC director 2018-2022) and Dr. Fortney (former AC co-director 2018-2022) will co-direct the pilot award and mentoring program. Both of these are well-developed and considered programs.
- The Administrative Core has been very collaborative with other ALACRITY Centers and has participated in junior faculty development both internally and externally.

- They have made a great website with helpful information about the Center and training videos.
- They already have an established R03 process, have awarded 12 grants through this process previously; a large number converted into other federal grants. These grants have been very multidisciplinary in nature.
- The Center as a whole has had a good publication record.
- There is a science and policy board as well as a stakeholder advisory board.
- The attention to equity, diversity, and inclusion (EDI) retention is appreciated, as well as recruitment for junior faculty. Also appreciated is the idea that investigators who contribute technology should get academic products that are valued in their fields. It seems there is a good commitment to equity here all-around.

Weaknesses

- It is not clear what the Training Hub was exactly.

Methods Core -1

Strengths

- 3 investigators co-lead the Methods Core: Drs. Lyon, Munson (also overall Center leads) and Pullman. Dr. Pullman is an experienced methodology mentor having previously led methodology foci/Cores for other NIH Center grants. There is also a good amount of full-time equivalent staff allocated to the Methods Core (MC). The MC monthly mentorship meetings are a strength.
- Overall, this was one of the strongest sections of the renewal. As mentioned in the “Overall” portion, the standardization of methods across projects is a strength, and many elements of this strategy are very well detailed in this Core (e.g., interventions, measures, analytic methodology). This will allow each project to learn from another and also UWAC 2.0 to learn from what was found in UWAC 1.0. The examples provided from specific projects were very helpful.
- The use of a shared conceptual model is also a strength.
- How they have mapped their new mechanisms to their modification targets is a strength. Also, a strength is the “library of redesign solutions”, which seems like it could have broad applicability.
- There are strong diversity, equity, and inclusion (DEI) considerations.
- The planned meta-analytic syntheses were a strong feature and add value to the Center as a whole.

Weaknesses

- Seems like there is a missed opportunity here to apply the DDBT paradigm to the redesign of UWAC2.

Research Projects

SIGNATURE PROJECT - 3

Strengths

- This project is an expansion of an R34 in the UWAC 1.0. It is a large-scale trial Hybrid Type 3 trial of PST-Aid versus PST-as-usual, two versions of problem-solving therapy, which is a CI for patients with depression. The intervention will be tested in the OCHIN network which is a very large network of community health centers. 60 practitioners from 20-30 clinics will participate. The primary outcomes of interest are implementation (fidelity, adoption, reach, reactive adaptations), DDBT mechanisms (usability, engagement, appropriateness), and patient outcomes (depression symptoms [Patient Health Questionnaire-9, PHQ-9], functioning [Quality of Life in Neurological Disorders Social Relations scale, Neuro-QOL]). There are also some “exploratory aims” looking at quality of goal setting and action planning as well as client task self-efficacy and behavioral activation. The project does a good job of measuring the mechanisms underlying the intervention effects. In addition, the comparison of the adapted intervention to the PST-as-usual is a strength since it focuses the study on CI/IS regarding PST, not PST per se.

- Overall, the project is of high significance since evidence-based CIs for depression are rarely implemented in primary care settings, despite the fact that depression is highly prevalent and has high morbidity and mortality. PST is an evidence-based treatment (EBT), but fidelity is usually low so it might be more effective if delivered with sustained higher fidelity. PST may be a poor cultural fit for diverse patients; it is argued that customized clinical decision support services (CDSS) may help improve fidelity and sustainability.
- They have preliminary data from their R34 showing that PST requires a pretty unreasonable amount of training and re-training, and also is implemented with low fidelity. There seemed to be particular problems with goal-setting. PST-Aid is a web-based app that provides more goal-setting scaffolding and support with the PST process generally, it had better adoption than the PST-as-usual in the pilot.
- In terms of innovation, this is similar to the overall Center in that looking at CI redesign for safety-net settings is fairly innovative. Studying the costs of the improved intervention is somewhat innovative. Overall, the innovativeness of this specific intervention might have been better articulated.
- The project is led by Drs. Bennett and Raue. Dr. Bennett is a family physician whose research focuses on mental health services implementation in non-specialty settings. He was the MPI of the prior R34, and has a current NIMH R01 involving the OCHIN network, and also is the PI of a semi-related R21. Dr. Raue is a Clinical Psychologist who was MPI with Dr. Bennett on the R34 as well as a Co-I on Dr. Bennett's R01. His research focuses on implementation of behavioral health interventions, particularly late in life. He is also an established investigator with an NIMH-funded R01 study regarding behavioral activation in senior centers. Overall, these investigators are well-qualified to lead this study. Dr. Lynch brings particular expertise in mental health implementation in the OCHIN network.
- The OCHIN network, which is quite large and diverse, is a strength of this research project. The quantitative data collection schedule for both providers and clients seems reasonable.
- The analytic process is clearly laid-out and seems appropriate.

Weaknesses

- It was difficult to get a feel for what the PST-Aid really "is" or involves. The screenshot was tiny and not very informative. It seemed that one had to trust them that what they had developed was actually better. There is concern that practitioners will be willing to use an app if this is not part of their usual workflow. Hopefully they have thought about this; it was not directly addressed. Moderate weakness.
- It also wasn't clear if PST-Aid integrates with OCHIN Epic, and if so whether OCHIN was willing to modify their Epic build for this project. As they know, it is hard for practitioners to get out of their electronic health record (EHR) environments in the clinic setting. Hopefully they have thought about this. Minor weakness.
- It was not clear whether PST-Aid took less time (or more time?) for practitioners to train on in the pilot. It seems that this could still be a barrier outside of the study context, even if subsequent fidelity is high. It is hard to imagine that many primary care based mental health practitioners have time for 20 hours of training. Minor/moderate weakness.
- Given that OCHIN has so many clinics, it is not clear how they will select clinics for inclusion. Minor weakness.
- It is not clear how the project will deal with the potential of intervention cross-contamination on the clinic level. Certainly providers will talk to one-another about the intervention. Minor weakness.
- There does not appear to be sufficient rationale provided for why the qualitative interviews need to be with every provider – this would be an excessive qualitative sample. Minor.
- It seems like attrition on the client and provider level should be measured as an outcome. Minor weakness.
- The costs analysis part of this project appears to be very under-developed. It is not clear what they were trying to measure or do. Minor/moderate weakness.

EXPLORATORY PROJECT – 001 - 6
Strengths

- The MPIs of this project are Dr. Locke and Dr. Bearss. Dr. Bearss is a child psychologist who focuses on evidence-based behavioral interventions for children with autism spectrum disorder (ASD). She is the PI of an NIMH R34 looking at RUBI in educational settings and also was PI of an R03 in UWAC 1.0 that provided pilot data for the other R34. Dr. Locke has a PhD in Education and is faculty in UW's psychiatry department. Her research focuses on interventions for autistic youth. She was the PI of a K01 and is the PI of two Institute of Education Sciences (IES) grants all on autism, as well as the R34 that Dr. Bearss is also on. Both MPIs have a history of collaboration and are well-prepared to lead this study.
- The topic of the project, implementation of CI for disruptive behaviors in ASD, is important and under-researched. It aligns well with the UWAC 2.0 mission as well as NIMH strategic priorities.
- The overall approach of DDBT makes sense for this product, and the user testing/ prototyping methods were strong.

Weaknesses

- This project had a very large number of organizational/grantsmanship issues that really impacted intelligibility. In particular, all the different versions of RUBI, RUBIES, RUBI IS, RUBIES IS, is really confusing—it does not help that they all have almost the same name. It was impossible to keep them straight in the renewal. It was hard to get past this issue in understanding the renewal as a whole, or why this additional study was necessary. Major weakness.
- Relatedly, the relationship between this R34, the other R34 that was not part of UWAC 1.0, and the prior R03 is confusing. It seems like there is substantial overlap; it was difficult to tell what was already made/adapted as part of which prior research, or what was new to this renewal. It is not clear if another pilot grant of this intervention is really needed. There have already been two (at least?). Major weakness.
- It was difficult to tell if this current project was different in that it focused on the needs of paraeducators (versus educators in general). This was very insufficiently explained. Moderate weakness.
- There is not enough explanation of what RUBI(ES) involves except that it says it is a manualized curriculum for disruptive behavior. It's therefore hard to understand whether it needs additional investment/adaptation. They finally get around to sort-of explaining it in the pilot trial (Aim 3) but by that point it seemed too late. Moderate weakness.
- Innovation seemed limited. As with the signature project, there did not seem to be a lot of innovativeness beyond the innovativeness of the Center as a whole, except perhaps for the digital design angle – which is not super innovative; this is definitely one of many recent efforts to use an app to improve evidence-based practice (EBP) use for ASD in schools. Minor/Moderate weakness.
- The "Local redesign team" didn't seem super local since it involves the whole research team. It is not clear how this is different from a "central redesign process". Minor weakness.
- 20 schools (180 people total) is quite a large sample for a pilot trial. It sounds like it would be a real organizational challenge, and it's not clear why so many would be needed if the goal is not to prove efficacy. It also was not clear how schools would be recruited. Also there is a concern that paraeducators will not have the time to commit to this intervention/training. Moderate weakness.

EXPLORATORY PROJECT – 002 - 2
Strengths:

- The goal of this project is to adapt trauma-focused CBT (TF-CBT) for the school setting (S-TF), and then to compare the original and adapted programs in a pilot hybrid type 2 randomized trial, assessing for both implementation outcomes (usability, engagement, appropriateness, adoption,

fidelity, reach, etc.) and student-level mental health mechanisms (trauma-related cognition, emotion regulation, behavioral avoidance) and mental health outcomes (symptoms of post-traumatic stress).

- The topic is timely given that many US youth experience trauma and the burden on youth mental health has increased in the Covid-19 pandemic. TF-CBT is used in schools but often with low fidelity. Some of the proposed possible modifications make sense.
- There is innovativeness in that there are not currently any TF-CBT interventions focusing on schools. The other factors mentioned are not really that innovative but rather just features of the study and Center as a whole.
- Dr. Lyon and Dr. Walker are MPIs. Dr. Lyon is also the overall Center director, and this type of intervention is in the area of his expertise. Dr. Walker is a clinical psychologist, an established investigator who has a somewhat eclectic portfolio and a recent focus on trauma-based interventions. Other co-I's bring complementary strengths. Study team is strong.
- Timeline seems reasonable.
- Good selection of measures. An especial strength are all the measures of research feasibility – this makes sense moving forward to an R01.
- There are letters of support (LOS) from participating school districts.

Weaknesses:

- Within districts, it is not clear how schools are selected for Study 1 and Study 2. Also, it is not clear what the age range of students is. It seems like it might be rather large (elementary through high schools participate; at one point it says age 3-18, in human subjects it seems to say 8-19). The needs of elementary schoolers and 18 year olds are really different (Also: it is not clear how 3-year-olds would even participate in the data collection). A narrower age range is needed, or there needs to be more tailored adaptation to younger versus older children (minor/moderate).
- It was not clear how many schools will be involved. It seems like it might be quite a lot, and this could be a lot of coordination (minor).
- In the pilot study, there might be study arm contamination at the school level; it is not clear how this will be addressed (minor).

EXPLORATORY PROJECT – 003 5

Strengths:

- This project is a hybrid type 3 pilot trial of BRISC, an intervention for a range of mental health problems in school settings. In the trial, the original BRISC program (BR-O) will be compared to a program that has been adapted for "diverse school settings" (BR-A). Primary outcomes will include implementation outcomes (similar to other project outcomes) and clinical outcomes (top problems, anxiety, depression, mental health [MH] functioning).
- Project of high significance given the need for MH interventions that work in schools, similar to project 002 above. In fact, there would be good synergy with project 002, which is a strength.
- The project is led by Drs. Bruns and McCauley. Dr. Bruns is a clinical psychologist with a focus on implementation science. It's unclear what his prior funding as PI is from his biosketch, but it seems adequate to lead this project since he has been involved in many other projects mostly not funded by NIMH. Dr. McCauley is a clinical psychologist who focuses on depression in youth. She has lots of experience in the school setting. This team is well-qualified.
- The methods are similar to the other projects and generally well-designed; appropriate outcomes selection. Sample size seems reasonable.

Weaknesses:

- This project differs from the others in that the original intervention was actually designed for implementation in schools. As is pointed out, it can be "implemented with fidelity, is feasible, increases use of EBP." It also seems like it is efficacious per data in a large-scale study. There is even an existing implementation strategy for BRISC. Thus it's not as justifiable why re-design is necessary. The argument that it needs to be re-designed for a targeted population might be

reasonable; however, it doesn't seem like there is a targeted population here since they are considering both lower- and higher-resource schools. "Diverse" is used really loosely here – it seems like this includes everyone.

- There also seems to be something about school counselors using this instead of other school mental health personnel, but this is not clearly articulated.
- The needs of the lower-resource and higher-resource schools might be really different, yet they are lumped together in the redesign process. It was hard to envision the end product(s) here. It is not clear if it is one redesigned BRISC or two different versions for different areas.
- Unlike other projects, they are not measuring the mechanisms of the mental health outcomes (e.g., emotion regulation, etc.). It was unclear why not. This is supposed to be included for all NIMH clinical trials.
- The innovation section of this project had 4 sentences. It does not seem like there is a commitment to innovation here.

Study Timeline - Overall
Comments:
- See above comments regarding milestones.

Protections for Human Subjects
Acceptable Risks and/or Adequate Protections
Data and Safety Monitoring Plan (Applicable for Clinical Trials Only):
- Acceptable

Inclusion Plans
- Sex/Gender: Distribution justified scientifically.
- Race/Ethnicity: Distribution justified scientifically.
- For NIH-Defined Phase III trials, Plans for valid design and analysis: Not applicable.
- Inclusion/Exclusion Based on Age: Distribution justified scientifically.

Vertebrate Animals
Not Applicable (No Vertebrate Animals)

Biohazards
Not Applicable (No Biohazards)

Applications from Foreign Organizations
Not Applicable (No Foreign Organizations)

Select Agents
Not Applicable (No Select Agents)

Resource Sharing Plans
Acceptable

Authentication of Key Biological and/or Chemical Resources
Not Applicable (No Relevant Resources)

Budget and Period of Support
Recommend as Requested

CRITIQUE 3

Significance: 2
Investigator(s): 2
Innovation: 2
Approach: 4
Environment: 2

Overall Impact:

The renewal is to continue work in integrating human centered design (HCD) with improvement in mental health services. The Center is proposing using similar models and methods but expanding to implement interventions in youth and school settings via the exploratory project. The Administrative and Methods Cores are strong in their combining approaches from Human-Computer Interaction (HCI) and implementation science. The main question mark of this renewal is the lack of discussing the shift of focus from adults in primary care to an increased emphasis on youth through exploratory projects that focus on autism, and trauma based cognitive behavioral therapy (CBT) school mental health interventions. There are merits to focusing on youth, but the shift or motivation was not expressly discussed. There are significant differences in the systems that these populations interface with and it might be helpful to consider those factors in the adaptation and implementation of interventions.

1. Significance:
Strengths
- Activities focus on underserved and needy populations.
- There is a strong structure to enable community-based work and applying the Center's Discover, Design/Build, Test (DDBT) framework across a variety of settings.

Weaknesses
- The focus has moved from adult populations to also include school based mental health. It might be helpful to make the connection about the similarities and differences of these settings.

2. Investigator(s):
Strengths
- The PIs provide complementary skill with one placing an emphasis implementation science in schools and the other in human centered design.

Weaknesses
- None noted by reviewer.

3. Innovation:
Strengths
- Expanding to youth mental health is important and needed.

Weaknesses
- On the flip side, of the strength in school mental health is the question of cohesion between the school work with the primary care work.

4. Approach:
Strengths
- The process of engaging in research projects is strong via an integration of design and services research that is cross disciplinary and necessary.

Weaknesses
- This shift is due to a change in leadership and thus reflects the expertise of the new PIs. It's understandable but the change in population focus wasn't addressed as directly as would be hoped for in order for the renewal to fit neatly together. This expansion is on the one hand a strength given the pressing need for youth mental health interventions, but it also presents a challenge to fit into the existing structure.

5. Environment:
Strengths
- Strong collaborations with primary care practices in a 5-state region via the Northwest Mental Health Technology Transfer Center.

Weaknesses
- The broader collaboration with school settings across a broad area isn't as strong as primary care.

Milestones:
Comments:
- The proposed milestones are feasible and developed and the record of previous success also bodes well.

Administrative Core
Strengths
- The Core is extremely strong in developing trainee success in publication and grants.
- There is an emphasis on recruiting and training underrepresented minority (URM) trainees.

Weaknesses
- The focus of the Center is moving to schools and youth, but the signature research project will focus on problem solving therapy in adults in primary care. It would have been helpful to discuss this shift and the implications to the Center's work. Perhaps we don't need to separate youth and adult work, but even that case wasn't made.

Methods Core
Strengths
- The Methods Core builds on previous work in identifying targets in implementation research and has been further adapted in this renewal application.
- Existence of measures in multiple languages and focus on a variety of populations.
- The methods of interest are clearly applied to the various projects that are proposed as part of the Center.
- Methods Core couples a strong emphasis on HCI with significant effort from the School of Engineering with behavioral science.

Weaknesses
- It is not clear if methods vary for adult versus school based populations.

Research Projects
Strengths
- Studying paraeducators is important to potentially scale interventions in school settings.

Weaknesses
- The target population is stated as underserved and people of color, but two of the schools being studied are in high resource/suburban areas. More information on how the larger goals are achieved in that setting would be important.

Study Timeline - Overall
Comments:
- Timelines are appropriate and feasible, particularly given previous experience.

Protections for Human Subjects
Acceptable Risks and/or Adequate Protections
- There are not concerns in this area.

Data and Safety Monitoring Plan (Applicable for Clinical Trials Only):

- Acceptable
  - The plan is clear and feasible with appropriate staff named and processes clearly delineated.

Inclusion Plans
- Sex/Gender: Distribution justified scientifically.
- Race/Ethnicity: Distribution justified scientifically.
- For NIH-Defined Phase III trials, Plans for valid design and analysis: Not applicable.
- Inclusion/Exclusion Based on Age: Distribution justified scientifically.
- There is school focus work which would include school aged populations and there is primary care work that focuses on adults.

Vertebrate Animals
Not Applicable (No Vertebrate Animals)

Biohazards
Not Applicable (No Biohazards)

Applications from Foreign Organizations
Not Applicable (No Foreign Organizations)

Select Agents
Not Applicable (No Select Agents)

Resource Sharing Plans
Acceptable
- The plan is clear and data will be shared with processes for sharing clearly delineated.

Authentication of Key Biological and/or Chemical Resources
Not Applicable (No Relevant Resources)

Budget and Period of Support
Recommend as Requested

CRITIQUE 4

Significance: 2
Investigator(s): 2
Innovation: 3
Approach: 4
Environment: 1

Overall Impact:
This submission of a P50 Advanced Laboratories for Accelerating the Reach and Impact of Treatments for Youth and Adults with Mental Illness (ALACRITY) Research Center renewal builds on the successes of an earlier Alacrity application and focuses on enhancing uptake of evidence-based psychosocial interventions in nonspecialty settings (i.e., schools, primary care settings). The focus is highly significant and has considerable potential. The investigative team, led by Dr. Lyon, is excellent and has the experience and expertise to run an effective and impactful Center. The Administrative and Methods Cores are solid and will provide clear and robust support to the ongoing work and research of the Center. The signature project and three exploratory projects have numerous strengths, although

their impact is tempered by various limitations. One overarching limitation is the lack of integration across the four studies. As one example, the three exploratory projects all focus on schools, while the signature project is located in primary care settings. Overall, the renewal is impressive in its scope, rigorous in its methods, and likely to make a notable impact.

1. Significance:
Strengths
- The focus on increasing uptake of evidence-based psychosocial interventions in nonspecialty settings is significant and a notable strength.
- Schools and primary care settings are ideal places to locate this work, both in terms of research and dissemination potential.
- The Center has considerable expertise in the range of relevant domains, making it likely that the work will be impactful.
- The Discover, Design/Build, and Test (DDBT) framework is well conceptualized and grounded in Human Centered Design (HCD). Moreover, it has been established through the team’s prior ALACRITY application and so is well-positioned to support the aims of this Center.
- The various studies have numerous strengths that will likely yield important information about enhancing service delivery in schools with regards to autism, trauma, and more, and then also in primary care.
- The focus on engagement, usability, and appropriateness as domains of implementation strategy (IS) is a notable strength.
- The Administrative and Methods Cores are well conceived to support the success of the Center.

Weaknesses
- Although the four studies have clear connections, there does not appear to be a clear coherence to them. They appear as distinct projects that don’t necessarily inform each other.
- The team’s prior ALACRITY center (UWAC 1.0) is referenced in indirect ways to highlight the experience the team brings, but not in direct ways. It is not clear to what extent UWAC 2.0 is building on the work of UWAC 1.0. For example, the projects seem quite distinct from those focused on in UWAC 1.0. Similarly, the leadership team is apparently different in notable ways. More information about the connections, or changes, between the two Center applications would be useful.
- Built into the philosophy of this approach is a recognition that the DDBT framework, by virtue of its data-driven approach, can provide minimal prediction regarding what actual adaptations that may emerge. Thus, it is plausible that the changes that emerge are of minimal and incremental value.
- Relatedly, it is unclear the extent to which modifications can address larger, systemic issues. Insofar as the settings are overburdened and underresourced, making minor changes to interventions may not yield the desired impact.

2. Investigator(s):
Strengths
- The team of investigators is excellent and well-positioned to effectively establish and run this Center. The PI, Dr. Aaron Lyon, has a strong record of both relevant research in increasing accessibility to psychosocial interventions in underresourced settings. He also has a strong record of administrative leadership as the previous director of the Methods Core in UWAC 1.0. He is supported in this work by the co-director of UWAC 2.0, Dr. Sean Munson, who is an expert in Human Centered Design and co-directed the Methods Core in UWAC 1.0. They are supported by co-investigators Drs. Areán and Fortney, who appear to have been previous leads of UWAC 1.0.
- The Methods Core directorship team brings considerable and relevant expertise. Dr. Michael Pullman has the requisite research background (mental health services and IS in child serving

systems) and the administrative/mentorship experience. Drs. Lyon and Munson have already co-lead the Methods Core for UWAC 1.0 and so bring a wealth of expertise to their roles.
- The leadership teams of the various projects are well-qualified, both in terms of research expertise and in terms of overseeing large grant-funded research.

Weaknesses
- The change in leadership team from UWAC 1.0 to UWAC 2.0 is significant and would benefit from some elaboration.
- Both Dr. Lyon and Dr. Munson are co-directors of the Administrative Core and also co-directors of the Methods Core. Dr. Lyon is also PI of one of the exploratory projects. This seems like a heavy lift.

3. Innovation:
Strengths
- The DDBT framework is moderately innovative, as is the use of HCD.

Weaknesses
- None noted by reviewer.

4. Approach:
Strengths
- The methodologies of the various projects are rigorous and well-considered to meet the overarching Center aims.
- The reach of the Recruitment timelines appears to be reasonable and feasible.
- Human subjects considerations have been carefully delineated, for both adolescents and clinicians.
- Stakeholder engagement has been bolstered.

Weaknesses
- Some concern that the sample sizes of the exploratory projects may preclude extensive exploration of data, but this is an understandable limitation.
- More generally, the integration of the different approaches is not well specified in the renewal.

5. Environment:
Strengths
- The facilities supporting this Center are excellent.
- The many collaborative relationships with schools and primary care settings appear to be long-standing and robust.

Weaknesses
- None noted by reviewer.

Milestones:
Comments:
- None noted by reviewer.

Administrative Core
Strengths
- Drs. Lyon and Munson seem well positioned to serve as co-directors of the Administrative Core, given their previous experience as co-directors of the Methods Core of UWAC 1.0. They will be supported by Drs. Areán and Fortney in this work. The additional team brings considerable experience to the work.
- The six enumerated aims are well considered for this Administrative Core. These include supporting community/stakeholder engagement, supporting synergy across research projects, supporting junior investigators through distribution of pilot funds and other mentoring, evaluating

Center progress, and disseminating findings. The likelihood of success in these six domains is high, given the overall team's experience with UWAC 1.0.
- The science and policy board, together with the stakeholder board, are comprised of scientific and practice experts who will support the work of the UWAC 2.0.
- The leadership structure appears to be thoughtfully considered to support good communication and decision-making.
- One notable strength is the explicit focus of using the pilot projects to enhance junior investigator careers, with a particular emphasis on investigators of color. The team appears to have created structures that go beyond just lip service to enhancing diversity.

Weaknesses
- The extensive list of accomplishments of UWAC 1.0 did not seem to note successes in the realm of diversity and investigators of color. This raises question about the extent to which the team has experience in this realm.

Methods Core
Strengths
- As noted above, the Methods Core directorship team brings considerable and relevant expertise.
- The accomplishments of the Methods Core of the UWAC 1.0 are numerous and impressive, and they set the stage nicely for advancing further in this work.
- The identification of IS modification targets/typology of appropriateness, usability, and engagement help focus the DDBT work. The library of design solutions is impressive and sets the stage for more effective work in UWAC 2.0.
- The Methods Core, by supporting the DDBT framework across all research projects, provides a consistent foundation for the work. This includes evaluation of adherence to DDBT processes and examining cost outcomes.
- Support in different methodological approaches beyond DDBT will support the implementation and evaluation of the different projects. The focus on diversity, equity, and inclusion (DEI) is also interesting and well considered.
- The evaluation and dissemination plans are appropriate.

Weaknesses
- As noted above, both Dr. Lyon and Dr. Munson have additional time-intensive roles, including as co-directors of the Administrative Core.
- It is unclear what expertise the MC has in attending to issues of DEI. More information here might have been useful.

Research Projects
Exploratory Projects:
Strengths
- Focusing on improving delivery of evidence-based intervention in schools (i.e., Research Units in Behavior Intervention [RUBI], Trauma-focused Cognitive Behavioral Therapy [TF-CBT], Brief Intervention for School Based Clinicians [BRISC]) for a range of common mental health conditions (i.e., disruptive behavior in children with autism, trauma, anxiety/depression) is a laudable goal and highly significant.
- The use of the DDBT framework to identify modifiable IS targets, redesign and evaluate the extant interventions has considerable potential. The iterative nature of the process, incorporating feedback from multiple stakeholders, increases the likelihood of identifying modifiable targets and developing an enhanced, more usable version of the interventions.
- Recruitment methods seem strong.
- Analytic plan incorporates mixed methods, including contextual analyses and stakeholder perspective.
- Dissemination strategies are excellent.

Weaknesses

- It is plausible that the modifications will be relatively minor and may not yield impactful differences in uptake. This is hard to evaluate given the unknown nature of the information gathered in the preliminary phases of the projects.
- Although the application notes that the studies are not adequately powered to properly compare symptom changes in students across interventions, the studies nevertheless position these aims centrally.
- There is some lack of information regarding enrolling students who are already receiving psychological services. It is not clear to what extent adding interventions might complicate evaluation of the intervention, as well as disrupt the treatment these students are already receiving.
- Although the overarching application notes the attention to underserved populations, and the HCD approach is conceivably sensitive to the nuances of these contexts, more explicit attention to context and culture might have strengthened the renewal.
- As noted above, the renewal understandably focuses its attention on users of the intervention, with the assumption that finding ways to enhance usability of the intervention will promote greater uptake. Less consideration has been given to the systemic factors that complicate uptake of these interventions (e.g., staffing numbers, school resources). This raises questions about the ultimate impact of any modifications.

Signature Project:
Strengths

- Problem Solving Therapy (PST) for depression in primary care has a demonstrated record of efficacy, and the application clearly documents challenges in clinician uptake.
- The focus on evaluating a clinical decision support system (CDSS) tool to support use of PST for depression has considerable potential. PST-Aid appears to directly address the reported obstacles to uptake of PST.
- In general, the preliminary studies set the stage nicely for the proposed research project.
- The hybrid effectiveness-implementation is methodologically rigorous. Use of mixed methods data collection and analyses is a notable strength. Addition of cost-analysis is an important element of the project.

Weaknesses

- The preliminary research on PST-Aid appears to have been evaluated with low numbers of participants.
- The application's focus on primary care settings that serve low-income, patients of color appears to be a change from the original settings in which PST-Aid was originally developed. Although the application describes local adaptation, it is plausible that the adaptations will be significant, raising the question of the need for additional piloting prior to large scale evaluation.

Study Timeline - Overall

Comments:

- None noted by reviewer.

Protections for Human Subjects
Acceptable Risks and/or Adequate Protections
Data and Safety Monitoring Plan (Applicable for Clinical Trials Only):

- Acceptable

Inclusion Plans

- Sex/Gender: Distribution justified scientifically.
- Race/Ethnicity: Distribution justified scientifically.
- For NIH-Defined Phase III trials, Plans for valid design and analysis: Not applicable.

- Inclusion/Exclusion Based on Age: Distribution justified scientifically.

Vertebrate Animals
Not Applicable (No Vertebrate Animals)

Biohazards
Not Applicable (No Biohazards)

Applications from Foreign Organizations
Not Applicable (No Foreign Organizations)

Select Agents
Not Applicable (No Select Agents)

Resource Sharing Plans
Acceptable

Authentication of Key Biological and/or Chemical Resources
Not Applicable (No Relevant Resources)

Budget and Period of Support
Recommend as Requested

CRITIQUE 5

Significance: 2
Investigator(s): 2
Innovation: 3
Approach: 4
Environment: 1

Overall Impact:
Psychosocial clinical interventions (CI) such as psychotherapy, counseling, and case management are a preferred mode of treatment by most people seeking care for mental health problems, particularly low-income, perinatal, ethnic/racial minority, and rural populations, but they are rarely available in settings where people in need seek care, such as "safety net" (i.e., primary care) clinics or schools. The central focus of the original P50 as well as the current renewal is to develop novel solutions to improve engagement with—as well as the usability and contextual appropriateness of—CI/ Implementation Strategies (IS) in lower sourced primary care clinics and schools. Over the last 4 years, the team developed an innovative design methodology (the Discover, Design/Build and Test framework [DDBT]). Over the five-year time frame, the team will conduct three R34 Projects devoted to the redesigning and testing CI/IS in schools, one R01 (in primary care settings), and up to 6 pilot studies.

This renewal application has many significant strengths, including a high and impactful level of productivity during the first award period, a team with highly complementary expertise with a long record of successful collaborations, an outstanding environment. They are some weaknesses, including perceived lack of synergy in components and some lack of analytic details (e.g., mediation analyses testing the target mechanism are underspecified and likely underpowered). Further, more attention to cost assessment might have been helpful. Finally, the pilot applications have adequate review and selection plans, but the renewal might have provided more details about the level of support these

applications will receive from the Methods Core (to ensure each of these projects is successful in leading to larger projects and advancing the goals of the Center).

1. Significance:
Strengths
- The proposed Center will address a critical need for improved mental health care in school, for which there is a substantial unmet need.
- If successful, this P50 renewal has the potential to provide critical information for developing novel interventions.

Weaknesses
- It is not clear that the Center is more than the sum of its parts. Not enough consideration of how each Project will synergize with the other projects and cores. While there are common themes across the projects (e.g., data collection includes the same set of core measures, etc.), each of the R34s and the R01 could stand alone as separate projects.
- The R01 will be conducted in a primary care setting; this is a distinct setting from all R34 (based in schools). There is a lack of integration of these components.
- The three R34 are somewhat repetitive (following the same template) and seem rather parallel than synergistic. Even the analytic plans seem to follow the exact same text, with minimal modifications.

2. Investigator(s):
Strengths
- These experienced scientists are eminently qualified to lead this research program.
- A good mix of established and junior investigators.

Weaknesses
- Biostatistics expertise is likely insufficient. Dr. Pullmann is tasked with everything data-wise (including qualitative analyses and supervising data management, in addition to co-leading the Methods Core).
- There seem to be changes in leadership and personnel from the first cycle, and this transition is not explicitly addressed.
- The team seems to be overcommitted, with investigators being responsible for multiple roles; the leaders of the Administrative core also are co-leaders of the Methods Core.

3. Innovation:
Strengths
- Research projects will collect a common core of outcomes on 1) CI/IS usability, engagement, and appropriateness for practitioners and clients; 2) implementation outcomes; and 3) client-reported outcomes.

Weaknesses
- This renewal represents incremental work by the study investigators; framework has been in place before. The renewal does not sufficiently address how UWAC 2.0 will differ from UWAC 1.0.
- The renewal suggests the potential for delivering cost-effective IS, yet there do not appear to be a focus on cost or cost-effectiveness.

4. Approach:
Strengths
- Study/project aims build on previous work from the first cycle by the study investigators.
- Stakeholders’ input was considered in designing all projects.
- Recruitment goals are feasible.
- Although outcomes are described, it is not always clear how they would be operationalized.
- Statistical approach can handle missing data and various degrees of clustering.

Weaknesses
- While the overall statistical approach is appropriate and clustering is considered, some of the details in statistical analysis plans and especially in the power analysis might have been clearer.
- Across projects, few alternative outcomes and challenges are discussed. Even if the investigators do not envision problems, some contingency plans might be useful.
- The scope of the individual projects (to include 3 pilot feasibility trials and 1 R01) are somewhat underwhelming given the size of the Center, and the fact that this is a renewal.
- None of the R34s from the first cycle appear to have led to R01 funding.

5. Environment:
Strengths
- Excellent and supportive environment.

Weaknesses
- None noted by reviewer.

Milestones:
Comments:
- Milestones seem appropriate and feasible within the specified timeframe.

Administrative Core
Strengths
- R03 pilot projects will follow a general procedure of call for proposals, rigorous NIH-style review, project monitoring and mentoring. Up to 6 pilots total (50k/year each) will be funded.
- Detailed information is provided on Center activities to support early-stage investigators (monthly mentoring, grant writers' boot Camp).
- Website for the Center provides educational materials for the broader research community.
- Previous leadership will be part in the pilot award team.
- Collaborations with other ALACRITY Centers.

Weaknesses
- It is not clear what level of support the pilot R03s will get from the Methods Core in terms of data management, recruitment, analysis, etc. No specific level of commitment appears to be provided, in either the Administrative or Methods Core.
- More clarity about the evaluation metrics to be used would be useful.
- The renewal notes an impressive 35 publications from the first cycle. Only 28 are referenced in The Administrative Core Publication List and some are Proceedings/Abstracts. Furthermore, some of the key personnel (e.g., Pullman) are not co-authors on any of these. Methods Core Progress Report Publication List has a larger list, but includes presentations, abstracts, and several "Under Review" that do not include the name of the journal, as well as several "in preparation". While the Submitted/In preparation show that the Center continues to be productive, it might have been helpful to have the publications delineated from the rest.

Methods Core
Strengths
- Methods Core faculty are well qualified to lead their respective components. They all have relevant record in the areas that will be pursued. In addition, many have a record of joint collaborations, both within and outside the prior P50.
- MC will provide data management for all Center studies.
- Standardization of methods across projects.

Weaknesses
- There are some concerns about the ability of the Core to offer adequate analytic statistical support to the Center's investigators. Overall, the statistical effort does not appear to be consistent with the scope of the Center. The Core notes that "Dr Pullman will supervise a full-

time PhD level research scientist to conduct analyses", but there did not appear to be a budget for such a person.
- Drs. Lyon and Munson also co-direct the Administrative Core.

Research Projects
Strengths
- Statistical analysis plans and power calculations are provided for all components.
- Clustering due to the provider is discussed.
- Use of qualitative analyses in all projects is a strength.
- For Exploratory Project 1, 2, and 3:
    - The aims R34 are centered around evaluating its feasibility and acceptability; clear benchmarks are proposed.
- For Signature Project:
    - Intraclass correlation coefficients (ICC) are considered for clustering, which is consistent with prior research.

Weaknesses
- For all Projects, the non-inferiority margins are presented in standardized units, without any discussion on what they translate to in terms of the Project specific outcomes.
- For Signature Project:
    - Randomization is at the practitioner level, but practitioners are nested within clinics. Thus, the same clinic may have practitioners in both arms. Contamination is not adequately discussed.
    - Pilot data is not adequately described and appears to be based on very small numbers (=3/group) and thus not reliable.
    - Although a power calculation is presented, it does not appear to be matching the analytic plan. For example, Poisson or logistic regression are listed as the analytic approach, but power is only presented assuming normality.
    - Although the project is based on an R34 from the first cycle, the effect sizes are not justified with pilot data, as appropriate. The effect sizes for the power calculation do not seem grounded in any preliminary data.
    - It is unclear if any missingness is being accounted for in analyses and power calculation for provider outcomes.
    - For Aim 1, the use of 1-sided testing is not justified. Even with 1-sided testing, the detectable effect sizes with a modest sample of 60 practitioners are quite large. It is not clear if all the clinician-level primary outcomes are expected to show this large effects.
    - For Aim 1, most of the provider outcomes are collected at 3 time points, but the analytic plan does not address this. Also, the primary endpoint is not clearly specified (6 month?) for these outcomes.
    - Power for testing the mediation model does not appear to be provided and it is unclear if the power is adequate to address this aim.
    - Although controlling for multiple comparisons is mentioned, the power calculations are not adjusting the type 1 error.
    - Cost analysis plan is lacking details.
- For Exploratory Project 1:
    - Although clustering due to the school is mentioned, it is not clear if power analyses account for it.
    - Assessing mediation in this pilot study is unlikely to yield meaningful results.
    - It is also not clear if the power analyses account for any attrition.
    - It is not clear what the noninferiority margin is based on.
- For Exploratory Project 2:
    - The same is true for this project: It is not clear what the noninferiority margin is based on.

  - Practitioners will be randomized; each will recruit 2 students. This strategy may lead to bias (versus randomly selecting 2 students from an eligible pool).
  - The analysis plan will ignore clustering. The strategy to dummy code the provider in order to account for clustering is not clear. Specific details should have been provided in Statistical Design and Power (the same issue is present in Project 1).
- For Exploratory Project 3:
  - In this project, 4 school districts will be randomized, but all the other participants (practitioners, students) will be recruited. This selection method may induce bias.
  - It is not clear what the noninferiority margin is based on and what it translates to for each outcome.

Study Timeline – Overall
Comments:
- Projected timeline seems feasible.

Protections for Human Subjects
Acceptable Risks and/or Adequate Protections
- Acceptable

Data and Safety Monitoring Plan (Applicable for Clinical Trials Only):
- Acceptable

Inclusion Plans
- Sex/Gender: Distribution justified scientifically.
- Race/Ethnicity: Distribution justified scientifically.
- For NIH-Defined Phase III trials, Plans for valid design and analysis: Not applicable.
- Inclusion/Exclusion Based on Age: Distribution justified scientifically.

Vertebrate Animals
Not Applicable (No Vertebrate Animals)

Biohazards
Not Applicable (No Biohazards)

Applications from Foreign Organizations
Not Applicable (No Foreign Organizations)

Select Agents
Not Applicable (No Select Agents)

Resource Sharing Plans
Acceptable

Authentication of Key Biological and/or Chemical Resources
Not Applicable (No Relevant Resources)

Budget and Period of Support
Budget Modifications Recommended (in amount/time)
Recommended budget modifications or possible overlap identified:
- There are some concerns about the ability of the Core to offer adequate analytic statistical support to the Center's investigators. Overall, the statistical effort is not consistent with the scope of the Center. The core notes that "Dr Pullman will supervise a full-time PhD level

research scientist to conduct analyses", but there did not appear to be a budget for such a person.

THE FOLLOWING SECTIONS WERE PREPARED BY THE SCIENTIFIC REVIEW OFFICER TO SUMMARIZE THE OUTCOME OF DISCUSSIONS OF THE REVIEW COMMITTEE, OR REVIEWERS' WRITTEN CRITIQUES, ON THE FOLLOWING ISSUES:

PROTECTION OF HUMAN SUBJECTS: ACCEPTABLE

INCLUSION OF WOMEN PLAN: ACCEPTABLE

INCLUSION OF MINORITIES PLAN: ACCEPTABLE

INCLUSION ACROSS THE LIFESPAN: ACCEPTABLE

COMMITTEE BUDGET RECOMMENDATIONS: Budget Modifications Recommended (in amount/time)
There are some concerns about the ability of the Core to offer adequate analytic statistical support to the Center's investigators. Overall, the statistical effort is not consistent with the scope of the Center. The core notes that "Dr Pullman will supervise a full-time PhD level research scientist to conduct analyses", but there did not appear to be a budget for such a person.

Footnotes for 2 P50 MH115837-05; PI Name: Lyon, Aaron Robert

NIH has modified its policy regarding the receipt of resubmissions (amended applications).See Guide Notice NOT-OD-18-197 at https://grants.nih.gov/grants/guide/notice-files/NOT-OD-18-197.html. The impact/priority score is calculated after discussion of an application by averaging the overall scores (1-9) given by all voting reviewers on the committee and multiplying by 10. The criterion scores are submitted prior to the meeting by the individual reviewers assigned to an application, and are not discussed specifically at the review meeting or calculated into the overall impact score. Some applications also receive a percentile ranking. For details on the review process, see http://grants.nih.gov/grants/peer_review_process.htm#scoring.
