# Supplementary material for: Harnessing Human-Centered Design for Evidence-Based Psychosocial Interventions and Implementation Strategies in Community Settings: Protocol for Redesign to Improve Usability, Engagement, and Appropriateness
Source: JMIR Res Protoc. 2025 Jan 29;14:e65446. doi: 10.2196/65446 (PMC11822321; doi:10.2196/65446)
Supplement: Multimedia Appendix 2 [file resprot_v14i1e65446_app2.docx]

Multimedia Appendix Table 1. Summary of University of Washington ALACRITY^a^ (Advanced Laboratories for Accelerating the Reach and Impact of Treatments for Youth and Adults with Mental Illness) Center (UWAC^b^) 2.0 projects and methods by Discover, Design and Build, Test (DDBT^c^) phase.

| Project^d^ | | Setting and participants by DDBT phase^d^ | Recruitment^d^ | Clinical Intervention being tested^d^ | Implementation strategy being tested^d^ | Study design^e^ and methods during UWAC 2.0 | Study design^e^ and methods during UWAC 1.0 |
| --- | --- | --- | --- | --- | --- | --- | --- |
| **R01^f^** | | | | | | | |
|  | PST^g^ -Aid  (April 2023 to April 2028) | Primary care clinics:   - Design and Build:   - 5 providers (ie, licensed/license-eligible therapists)   - 4 clients - Test:   - 60 providers (ie, licensed/license-eligible therapists)   - 350 clients | - Providers recruited via organization membership - Clients recruited by their provider on the basis of the inclusion criteria | - PST (evidence-based, skills-based CI for depression that teaches clients a 7-step approach) | - PST-Aid (web-based app that promotes provider–client collaboration in the use of PST for goal setting and action planning) | - Discover: activities completed during UWAC 1.0 serve as the “Discover” phase for UWAC 2.0 - Design/build: observation, co-design, prototyping, and think-aloud protocol - Test: randomized controlled hybrid type 3 effectiveness-implementation trial with observation (audio recordings of sessions), interviews, and surveys | - Discover: interviews, observations of new and experienced PST trainers, and affinity diagramming - Design/build: prototyping and co-design of potential products to enhance fidelity when conducting PST - Test: pilot randomized trial comparing PST training-as-usual to training plus PST-Aid |
| **R34^f^** | | | | | | | |
|  | RUBIES^h^  (April 2023 to April 2026) | Schools:   - Discover:   - 10 paraeducators   - 14 school personnel (eg, principals and teachers) - Design and Build:   - 22 educators - Test:   - 80 educators   - 80 students with autism | - Elementary schools with students with autism will be identified via school district officials. School principals will then identify educators/school personnel and students will be identified by the educators | - RUBIES protocol (low-intensity manualized intervention for children with autism with co-occurring disruptive behavior, originally developed for parents and redesigned for educators) | - RUBIES-Team IS (emphasizes team collaboration and offers supplemental RUBIES resources) | - Discover: focus groups, cognitive walkthrough for implementation strategies, think-aloud protocol, interviews, and surveys - Design and Build: prototyping and user testing - Test: randomized hybrid type 3 effectiveness- implementation pilot trial | - Discover: in-class behavioral observations, retrospective cognitive walkthroughs, interviews, demonstration of the original Research Units in Behavioral Intervention paired with behavioral rehearsal, prospective think-aloud, and structured assessment methods to identify targets for Research Units in Behavioral Intervention redesign - Design and Build: prototyping of RUBI content on the basis of the identified targets for redesign - Test: N/A^i^ |
|  | TF-CBT^j^ (April 2024 to April 2027) | Schools:   - Discover, Design and Build:   - 15 school-based mental health practitioners   - 15 students - Test:   - 24 school-based mental health practitioners   - 48 students | - Practitioners will be identified via school principals - Students will be recruited from practitioner caseloads and broader study advertisement | - TF-CBT (a clinical intervention for children impacted by trauma and their caregivers adapting for school-based practitioner use) | - N/A (adapting intervention only) | - Discover: contextual evaluation, think-aloud protocol, behavioral rehearsal, interviews, and user testing - Design and Build: prototyping and user testing - Test: randomized hybrid type 2 effectiveness-implementation pilot trial | - Discover: N/A - Design and Build: N/A - Test: N/A |
|  | BRISC^k^  (April 2025 to April 2028) | Schools:   - Discover, Design and Build:   - 6 school-based mental health practitioners - Test:   - 12 school-based mental health practitioner   - 48 students | - Practitioners will be identified via school principals - Students will be recruited from practitioner caseloads | - BRISC (4-session engagement, brief intervention, and triage strategy targeting a range of mental health and other problems) | - BRISC implementation strategy package adapted to local needs (for use by school-based providers) | - Discover: observation, Cognitive Walkthrough for Implementation Strategies, think-aloud protocol, interviews, focus groups, and surveys - Design and Build: co-design and user testing - Test: randomized hybrid type 3 effectiveness-implementation pilot trial | - Discover: N/A - Design and Build: N/A - Test: N/A |

^a^ALACRITY: Advanced laboratories for accelerating the reach and impact of treatments for youth and adults with mental illness.

^b^University of Washington ALACRITY.

^c^DDBT: Discover, Design and Build, Test.

^d^Project details refer to UWAC 2.0.

^e^All study designs are in the context of a DDBT approach.

^f^All studies used convenience sampling methods.

^g^PST: Problem Solving Treatment.

^h^RUBIES: Research Units on Behavioral Intervention in Educational Settings.

^i^N/A: not applicable.

^j^TF-CBT: Trauma-Focused Cognitive Behavioral Therapy

^k^BRISC: Brief Intervention Strategy for School Clinicians
