# Supplementary material for: Harnessing Human-Centered Design for Evidence-Based Psychosocial Interventions and Implementation Strategies in Community Settings: Protocol for Redesign to Improve Usability, Engagement, and Appropriateness
Source: JMIR Res Protoc. 2025 Jan 29;14:e65446. doi: 10.2196/65446 (PMC11822321; doi:10.2196/65446)
Supplement: Multimedia Appendix 3 [file resprot_v14i1e65446_app3.docx]

**Multimedia Appendix 3.** Center Measures and Guidance.^a^

| Construct | | Measure/activity | Phase | | |
| --- | --- | --- | --- | --- | --- |
|  | |  | Discover | Design and Build | Test |
|  | | | | | |
| **DDBT^b^ mechanisms** | | | | | |
|  | **Usability** | | | | |
|  |  | Usability issues grounded in participant data and reported using UWAC’s^c^ standard structure | Yes | Yes (if design phase identifies further usability issues with either the existing clinical intervention or implementation strategy or redesigned one) | Yes (if design phase identifies further usability issues with either the existing clinical intervention or implementation strategy or redesigned one) |
|  |  | System Usability Scale, Intervention Usability Scale, or Implementation Strategy Usability Scale | Yes (existing clinical intervention or implementation strategy) | Yes (redesign clinical intervention or implementation strategy) | Yes (redesign clinical intervention or implementation strategy) |
|  | **Engagement** | | | | |
|  |  | User Responsiveness Scale | Yes (existing clinical intervention or implementation strategy if it exists and is possible) | Maybe (redesign clinical intervention or implementation strategy) | Yes (redesign clinical intervention or implementation strategy) |
|  |  | Coding of qualitative interactions | Yes (existing clinical intervention or implementation strategy) | Maybe (redesign clinical intervention or implementation strategy) | Yes (redesign clinical intervention or implementation strategy) |
|  | **Appropriateness** | | | | |
|  |  | Intervention Appropriateness Measure | Yes (existing clinical intervention or implementation strategy) or based on existing literature | Maybe (redesign clinical intervention or implementation strategy) | Yes (redesign clinical intervention or implementation strategy) |
|  |  | Revised goodness of fit interview | Yes (existing clinical intervention or implementation strategy);  optional (R03) | Maybe (redesign clinical intervention or implementation strategy);  optional (R03) | Yes (redesign clinical intervention or implementation strategy);  optional (R03) |
| **Proximal implementation outcomes** | | | | | |
|  | **Adoption and reach** | | | | |
|  |  | User report | Yes (existing clinical intervention or implementation strategy) or based on existing literature | No | Yes |
|  | **Intervention and implementation strategy fidelity** | | | | |
|  |  | Fidelity of practice | Yes (existing clinical intervention or implementation strategy) or based on existing literature | No | Yes |
|  | **Planned adaptations (ie, redesign solutions)** | | | | |
|  |  | Framework for reporting adaptations and modifications to evidence-based interventions and implementation strategies | No | Yes | Yes |
|  | **Unplanned or reactive modifications** | | | | |
|  |  | Framework for reporting adaptations and modifications to evidence-based practices | No | No | Yes |
| **Distal service recipient outcomes** | | | | | |
|  | **Client outcomes** | | | | |
|  |  | Quality of life in neurological disorders | No | No | Yes |
|  |  | Top problems assessment | No | No | Yes |
|  |  | Diagnostic and statistical manual of mental disorders, fifth edition level 1 cross-cutting symptom measure | No | No | Yes;  optional (R03) |
|  |  | Revised children’s anxiety and depression scale-25 | No | No | Yes;  optional (R03) |
|  |  | Patient health questionnaire-9 | No | No | Yes;  optional (R03) |
|  |  | General anxiety disorder-7 | No | No | Yes;  optional (R03) |
|  |  | WHO^d^ disability assessment schedule | No | No | Yes;  optional (R03) |
| **Demographic and process measures** | | | | | |
|  | **Demographics** | | | | |
|  |  | Participant demographics | Yes | Yes | Yes |
|  | **User needs and experience** | | At least one | At least one | At least one |
|  |  | User interviews |  |  |  |
|  |  | User-centered design activities |  |  |  |
|  |  | Other methods for understanding and probing user needs |  |  |  |
|  | **Participant research burden, incentive appropriateness, and research satisfaction** | | | | |
|  |  | 3 study-specific items, based on insights from the Conducting Research to Enhance Assessment and Treatment through Innovation in Mental Health Lab’s ADAPT: Message-Based Psychotherapy and Digital Treatment Sequences for Depression study pilot trial [83] | No | No | Yes |
|  | **Adherence to DDBT process (DDBT fidelity and cost measure)** | | | | |
|  |  | Survey; optional (R03) | Yes; optional (R03) | Yes;  optional (R03) | Yes;  optional (R03) |
|  | **Team collaboration, trust, and respect** | | | | |
|  |  | Transdisciplinary tobacco use research center satisfaction measure of team collaboration and transdisciplinary integration (productivity and satisfaction sections only or process quality and outcomes) | Yes; optional (R03) | Yes ; optional (R03) | Yes; optional (R03) |
|  | **Community participation in research** | | | | |
|  |  | Modified ladder of participation measure | Yes ; optional (R03) | Yes ; optional (R03) | Yes; optional (R03) |
|  | **Investigator satisfaction with the support they receive from the center** | | | | |
|  |  | UWAC satisfaction measure | N/A^e^ | N/A | N/A |

^a^Not all teams may complete all phases (particularly among smaller-scale pilot projects), and some pilot projects may begin with Design and Build phase when prior work by the project team or in literature sufficiently informs the design phase.

^b^DDBT: Discover, Design and Build, and Test.

^c^UWAC: University of Washington ALACRITY (advanced laboratories for accelerating the reach and impact of treatments for youth and adults with mental illness) Center.

^d^WHO: World Health Organization.

^e^N/A: not applicable.
